# Supplementary material for: A Wolbachia factor for male killing in lepidopteran insects
Source: Nat Commun. 2022 Nov 14;13:6764. doi: 10.1038/s41467-022-34488-y (PMC9663696; doi:10.1038/s41467-022-34488-y)
Supplement: Supplementary file 1 — Supplementary Information [file 41467_2022_34488_MOESM1_ESM.pdf]

Supplementary Information for:

# A *Wolbachia* factor for male killing in lepidopteran insects

Susumu Katsuma[1,5*], Kanako Hirota[1,5], Noriko Matsuda-Imai[1,5], Takahiro Fukui[1], Tomohiro Muro[1], Kohei Nishino[2], Hidetaka Kosako[2], Keisuke Shoji[3], Hideki Takanashi[1], Takeshi Fujii[4], Shin-ichi Arimura[1], and Takashi Kiuchi[1]

[1]Department of Agricultural and Environmental Biology, Graduate School of Agricultural and Life Sciences, The University of Tokyo, 1-1-1 Yayoi, Bunkyo-ku, Tokyo 113-8657, Japan
[2]Division of Cell Signaling, Fujii Memorial Institute of Medical Sciences, Institute of Advanced Medical Sciences, Tokushima University, Tokushima 770-8503, Japan
[3]Institute for Quantitative Biosciences, The University of Tokyo, 1-1-1 Yayoi, Bunkyo-ku, Tokyo 113-0032, Japan
[4]Faculty of Agriculture, Setsunan University, Hirakata, 45-1 Osaka 573-0101, Japan
[5]These authors should be regarded as joint first authors.

*Corresponding author. Mailing address: Department of Agricultural and Environmental Biology, Graduate School of Agricultural and Life Sciences, University of Tokyo, Yayoi 1-1-1, Bunkyo-ku, Tokyo 113-8657, Japan
Phone: 81-3-5841-8994. Fax: 81-3-5841-8993. E-mail: skatsuma@g.ecc.u-tokyo.ac.jp

Running title: A *Wolbachia* factor for male killing in moths

Supplementary Fig. 1

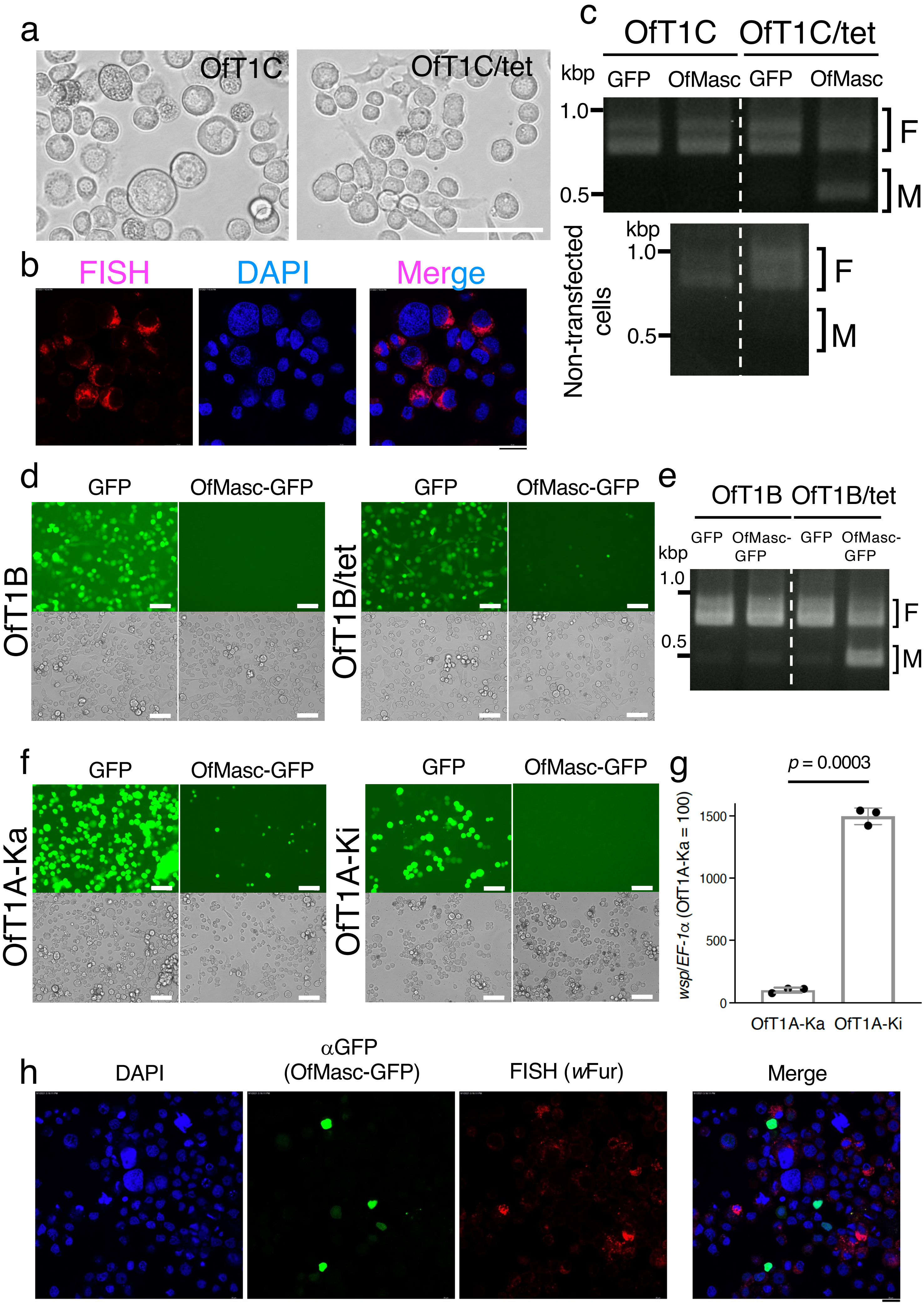

**<u>Supplementary Fig. 1. Characterization of *w*Fur-infected *O. furnacalis* cell lines.</u>**

a. Light microscopy of OfT1C and OfT1C/tet cells. Bar, 100 µm. Similar results were obtained in two independent experiments.

b. FISH analysis of *Wolbachia* in OfT1C cells. *Wolbachia* were detected by a 16S rRNA FISH probe. The cells were also stained with DAPI for DNA. Bar, 20 µm. Similar results were obtained in two independent experiments.

c. Splicing patterns of *Ofdsx*. OfT1C and OfT1C/tet cells were transfected with *GFP* or *OfMasc* cDNA, and the splicing patterns of *Ofdsx* were investigated. Splicing patterns in non-transfected cells are also shown. The F and M indicate female- and male-type splicing of *Ofdsx*, respectively. Similar results were obtained in two independent experiments.

d. Fluorescence microscopy of OfT1B and OfT1B/tet cells transfected with *GFP* or *OfMasc-GFP* cDNA. Bar, 100 µm. Similar results were obtained in two independent experiments.

e. Splicing patterns of *Ofdsx*. OfT1B and OfT1B/tet cells were transfected with *GFP* or *OfMasc-GFP* cDNA, and the splicing patterns of *Ofdsx* were investigated. The F and M indicate female- and male-type splicing of *Ofdsx*, respectively. Similar results were obtained in two independent experiments.

f. Fluorescence microscopy of OfT1A-Ka and OfT1A-Ki cells transfected with *GFP* or *OfMasc-GFP* cDNAs. Bar, 100 µm. Similar results were obtained in two independent experiments.

g. *Wolbachia* density in OfT1A-Ka and OfT1A-Ki cells. *Wolbachia* density was estimated by qPCR of *wsp* and normalized by *EF-1*$\alpha$. Data shown are means ± SD of analyses performed in triplicate. Similar results were obtained in two independent experiments. $p = 0.0003$, unpaired $t$ test (two-tailed) with Welch's correction.

h. Immunofluorescence analysis of OfMasc-GFP in OfT1C cells. *w*Fur was detected by FISH and the cells were also stained with DAPI for DNA. Bar, 20 µm. Similar results were obtained in two independent experiments.

Source data are provided as a Source Data file.

Supplementary Fig. 2

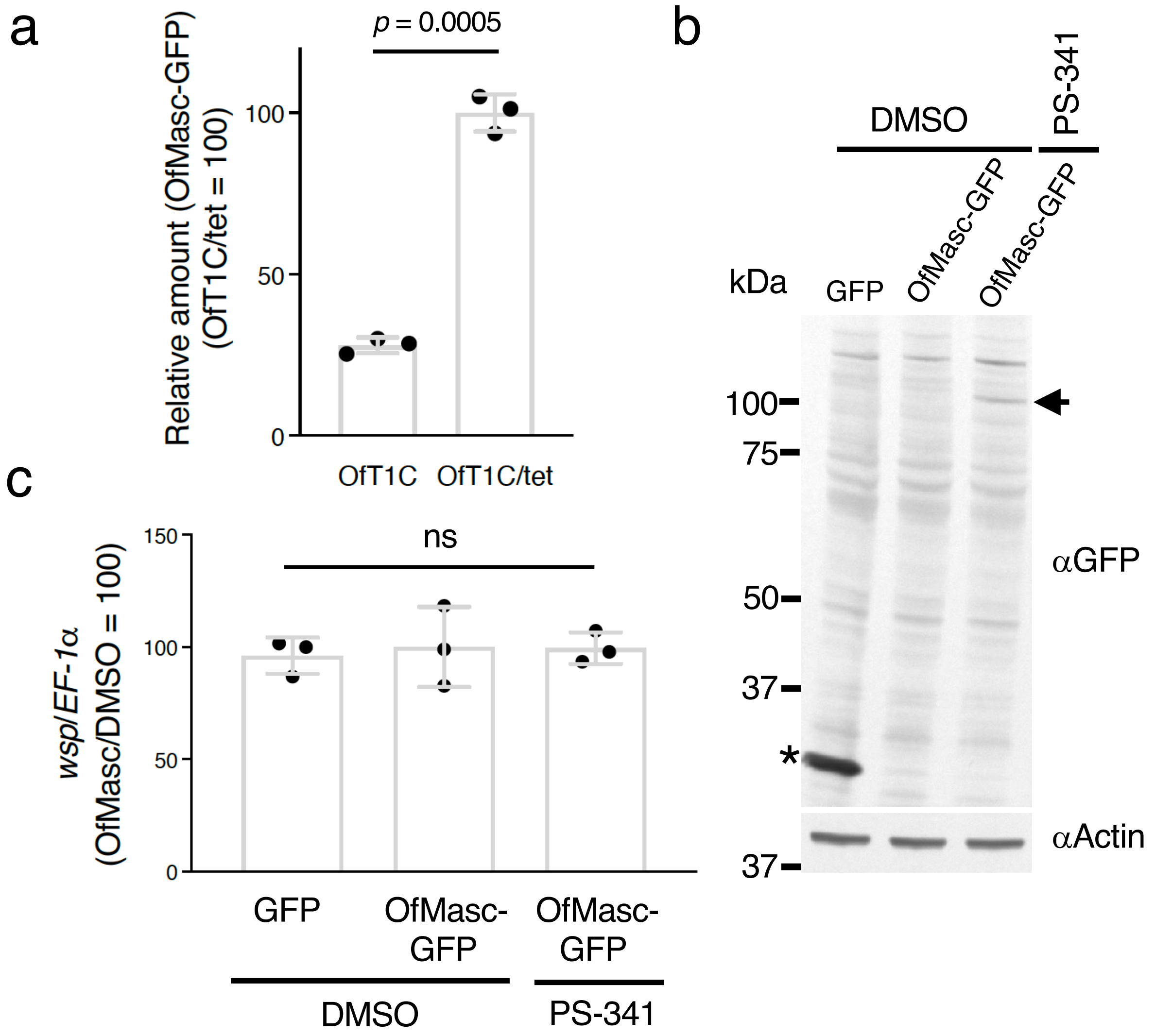


**<u>Supplementary Fig. 2. Reduced accumulation of OfMasc in *w*Fur-infected OfT1C cells.</u>**
a. Accumulation of OfMasc-GFP in OfT1C and OfT1C/tet cells. The intensities of OfMasc-GFP bands were measured and normalized with those of GFP. Data shown are means ± SD of data from three independent experiments. $p = 0.0005$, unpaired $t$ test (two-tailed) with Welch's correction.
b. Immunoblots of GFP and OfMasc-GFP in OfT1C cells treated with dimethyl sulfoxide (DMSO) or PS-341. Cells were transfected with *GFP*- or *OfMasc-GFP*-expressing plasmid, treated with DMSO or PS-341 (200 nM) at 2 days after transfection, and harvested for immunoblotting at 3 days after transfection. The arrow and asterisk indicate the positions of OfMasc-GFP and GFP, respectively. Actin was used as a control. Similar results were obtained in two independent experiments.
c. *Wolbachia* density in OfT1C cells treated with DMSO or PS-341 (200 nM). *Wolbachia* density was estimated by qPCR of *wsp* and normalized by *EF-1α*. Data shown are means ± SD of analyses performed in triplicate. Similar results were obtained in two independent experiments. ns, $p > 0.05$, one-way ANOVA.
Source data are provided as a Source Data file.

# Supplementary Fig. 3

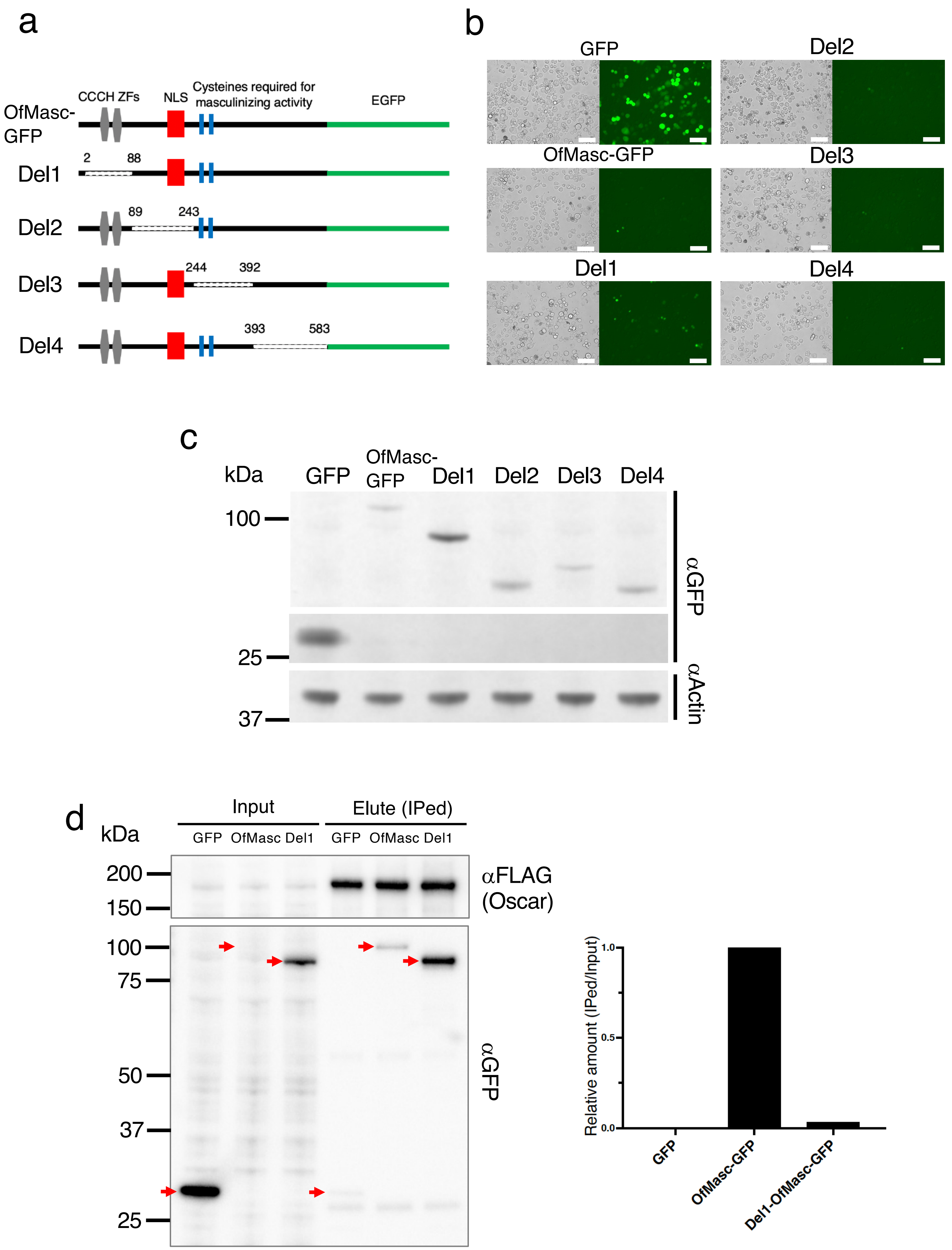

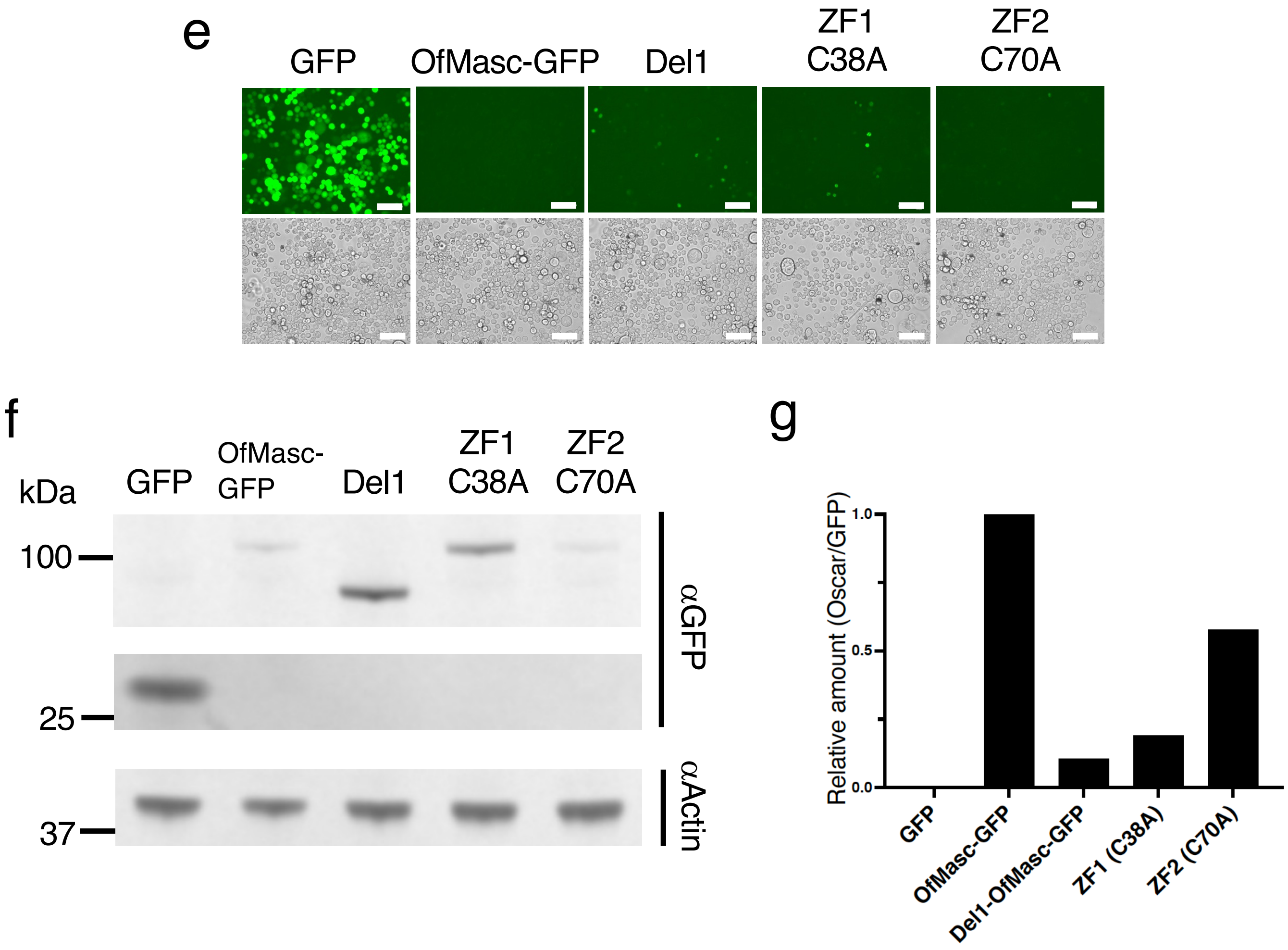


**<u>Supplementary Fig. 3. Identification of OfMasc regions that interact with the *w*Fur factor.</u>**

a. Structure of OfMasc-GFP derivatives.

b. Fluorescence microscopy of OfT1C cells transfected with *GFP*, *OfMasc-GFP*, *Del1*, *Del2*, *Del3* or *Del4* cDNAs. Bar, 100 µm. Similar results were obtained in two independent experiments.

c. Immunoblots of GFP, OfMasc-GFP, and its derivatives (Del1, Del2, Del3 or Del4) in OfT1C cells. Actin was used as a control. Similar results were obtained in two independent experiments.

d. Co-immunoprecipitation experiments of OfT1C/tet cells co-transfected with *3×FLAG-Oscar* and *GFP*, *OfMasc-GFP or Del1-OfMasc-GFP.* The immunoprecipitates with anti-FLAG antibody beads were immunoblotted using anti-FLAG or anti-GFP antibody. Relative amount of Oscar-bound GFP, OfMasc-GFP, or Del1-OfMasc-GFP is shown as IPed/Input. The measured bands are indicated by red arrows. Similar results were obtained in two independent experiments.

e. Fluorescence microscopy of OfT1C cells transfected with *GFP*, *OfMasc-GFP*, *Del1*, *ZF1 (C38A)* or *ZF2 (C70A)* cDNAs. Bar, 100 µm. Similar results were obtained in two independent experiments.

f. Immunoblots of GFP, OfMasc-GFP, and its derivatives (Del1, ZF1 or ZF2) in OfT1C cells. Actin was used as a control. Similar results were obtained in two independent experiments.

g. PRM quantification of relative amount of Oscar to GFP in *GFP*-, *OfMasc-GFP*-, *Del1-OfMasc-GFP*, *ZF1-OfMasc-GFP*- or *ZF2-OfMasc-GFP*-transfected OfT1C cells. Similar results were obtained in two independent experiments.

Source data are provided as a Source Data file.

# Supplementary Fig. 4

MEDRHIPFLSSLAILLWYGINKADRYGYTPLHLAVQSGNLEIVNALVGRGADVDKADRY
(ANK1)
ERTPLHLAVQSGNLEIINALVGRGADVDKADRYERTPLHLAVQSGNLEIINALVGRGAD
(ANK2) (ANK3)
VNKADKYGSTPLNFAAQIGNAEIINALVAHEVGDDICTPLHLAVLRNDMELVNSLIKQG
(ANK4) (ANK5)
ANVCQESRGRYIPLHLAARAGNVEMINALIEQGAKINQMNNDRFTPLHFAVQVGDVDVV
(ANK6) (ANK7)
NALLARSANVNEVGKYGFTPLHLATQVSNAEVIRALIERGADVNKADKYGYTPLHLAVQ
(ANK8) (ANK9)
SGNLEIINALVGRGADINKADKYERAPLHLAVQVGDVNVVNALLARSANVNKVDRYGHT
(ANK10) (ANK11)
PLHLAVQSGNLEIINALVGRGADINKADKYERAPLHLAVQVGDVNVVNALLARSANVNK
(ANK12)
VDRYGHTPLHLAVQSGNLEIINALVGRGADINKADKYERAPLHLAVQVGDVNVVNALLA
(ANK13) (ANK14)
RSANVNKVDRYGHTPLHLAVQSGNLEIINALVGRGADINKADKYERAPLHLAVQVGDVN
(ANK15) (ANK16)
VVNALVGRGADINKADKYERAPLHLAVQVGDVNVVNALLARSANVNKVDRYGHTPLHLA
(ANK17) (ANK18)
VQSGNLEIINALVGRGADVNKADKYGSTPLNFAAQIGNAEIINALVAHEVGDDICTPLH
(ANK19) (ANK20)
LAVLRNDMELVNSLIKQGANVCQESRGRYIPLHLAARAGNVEMINALIEQGAKINQMNN
(ANK21)
DGFTPLHFAVQVGDVDVVNALLARSANVNEVGKYGFTPLHFAAQVSNAEVIRALIERGA
(ANK22) (ANK23)
GVNQMNSDESVFGSSDRYAYGSTPLHLAIEKGNFEAVNCLLEEGADINQTDGYGRTPLH
(ANK24) (ANK25)
LAIEEGNFEAVNALVGRGADVNKADRYERTPLYLAVQLGNLEMVNALVGRGADINKADK
(ANK26)
YERTPLNLAVQLGNAEIINALIAHEVGDDICTPLHLAVLRNDMELVNSLIERGVDVNKA
(ANK27) (ANK28)
NEYGFTPLHFAIERGNFDAVNALISHQVGDDDRYTPLHLAVLRKNDIGLVNSLIEQGAD
(ANK29) (ANK30)
VDEVSRDRYTPLHLAVRSNNIQMVDTLIKRGANVNRINRDGYTPLHLAVQVGNVELINA
(ANK31) (ANK32)
LIKGSANINKVDRHGHTVLHLAIKSNNIQVVNALIERGVDVNKANRYGFTPLHFAVRSN
(ANK33) (ANK34)
NIQMVDTLIKRGANVNRINRDGYTPLHLAVQVGNVELINALIKGSANINKVDRHGHTVL
(ANK35) (ANK36)
HLAIKSNNIQVVNALTERGADVNKADRYGFTPLHFAVERGNFEVVDTLIKRGADVNRVG
(ANK37)
RRGHTALHWAIRENNIQVVNALIERGADVNRVGRRGHTALHWAIRENNIQVVNALIERG
(ANK38) (ANK39)
ADVNRVSRSGHTVLHWAVKSNNIQAVNTLIEQGAEIKDVDYPLDWAVGYEFQDIAKLLM
(ANK40)
EHEVKLKGCNVRKPRDIVQHNPDLEIEASEYIDRCVNEIEGIKKEIINGNVTFYDLLTS
KGDKLASLMLNKDIVTVLEKGEYKSKFPTYSKIMESQYEIGKENLHLFNRALEAIFNLF
VVKNLPLEIGEMIVKYLPSSFDRKSLIKLVEVKGLPNEKVIERRSSLEHKTIPTTQTVQ
DILTQIRGILDNQQIEVNDLLTLEFATSAGYYDYWLQQIDIARAARLLYQFCTKGNHTF
EVANLEGNDGNEPIVGVLNQFRENEEQQRLTLIITLNNAHWVTLVIERQNGNYVGYYAD
STATAVPGDITDIIQNNLGNNIRINNVSVSQQTDGWNCGLWALENANSINRVLNENPVG
KVQNIINIIRDFLERGHPKRDRNYFQNIRVGISQLFRNDPGFQDVQLQAYIQNREQIDP
LLRTYLELGYHRVGGGYGLVEAPISLGVSDPSNFGDLLQSSLEGPEASRVSDLSKGKGG
R
(CifB C-terminus-like)

**Supplementary Fig. 4. Amino acid sequence and domain structure of the Oscar protein.**
Ankyrin repeats and CifB C-terminus-like domain in the Oscar protein are shown.

Supplementary Fig. 5

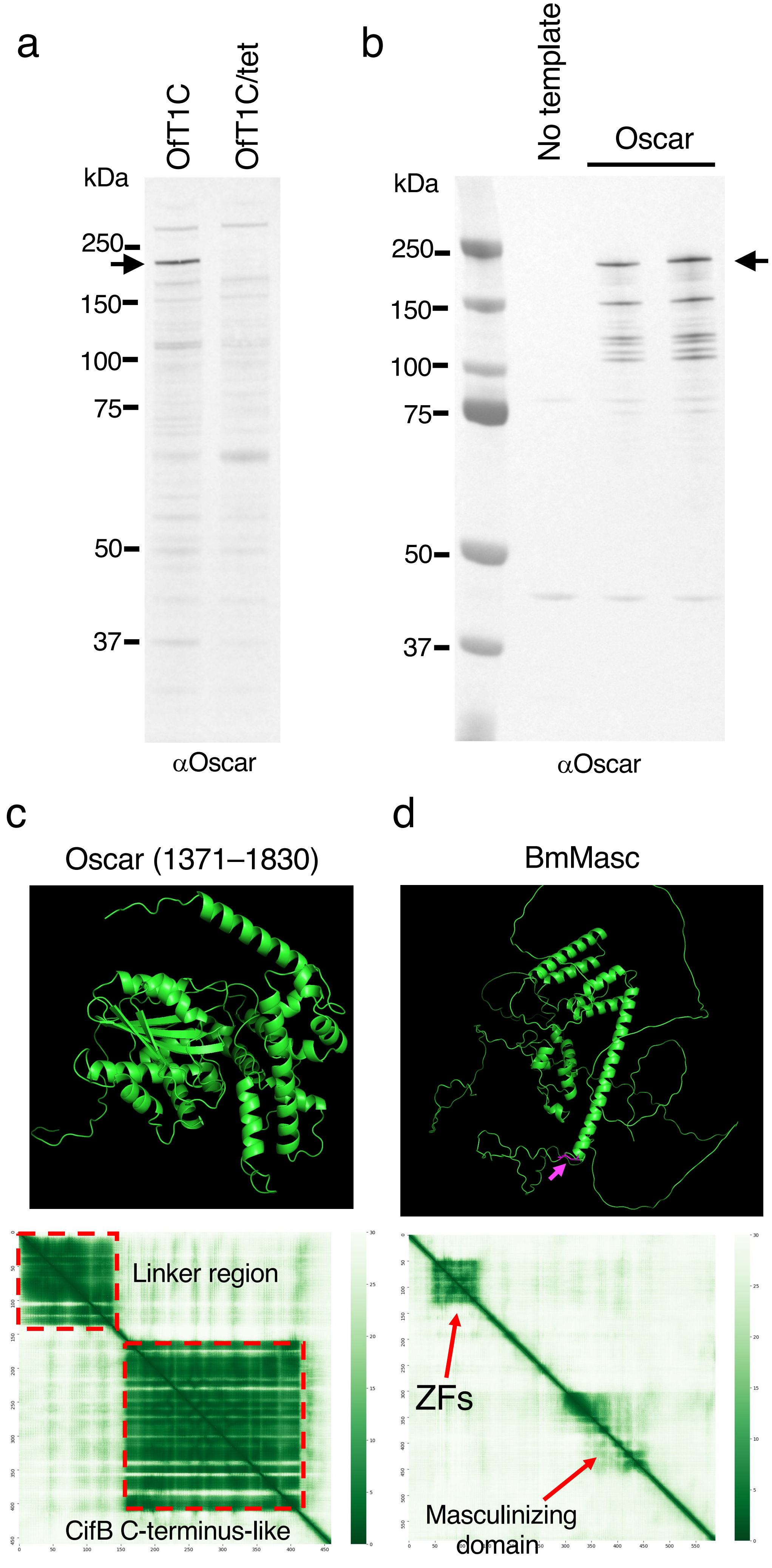

**Supplementary Fig. 5. Expression of Oscar and predicted structures of BmMasc and Oscar CifB C-terminus region.**

a. Detection of endogenous Oscar protein in OfT1C and OfT1C/tet cells by immunoblotting with anti-Oscar antibody. Arrow indicates the position of Oscar. Similar results were obtained in two independent experiments.

b. Production of recombinant Oscar protein by *in vitro* translation. Recombinant Oscar protein was detected by immunoblotting with anti-Oscar antibody. Arrow indicates the position of Oscar. Similar results were obtained in two independent experiments.

c. Structure of Oscar's linker/CifB C-terminus region (a.a. 1,371–1,830) predicted by AlphaFold2. The heatmap of predicted aligned errors is shown below the model.

d. Structure of BmMasc predicted by AlphaFold2. Two cysteine residues at 301 and 304 are shown in magenta (also indicated by magenta arrow). The heatmap of predicted aligned errors is also shown below the model.

Source data are provided as a Source Data file.

# Supplementary Fig. 6

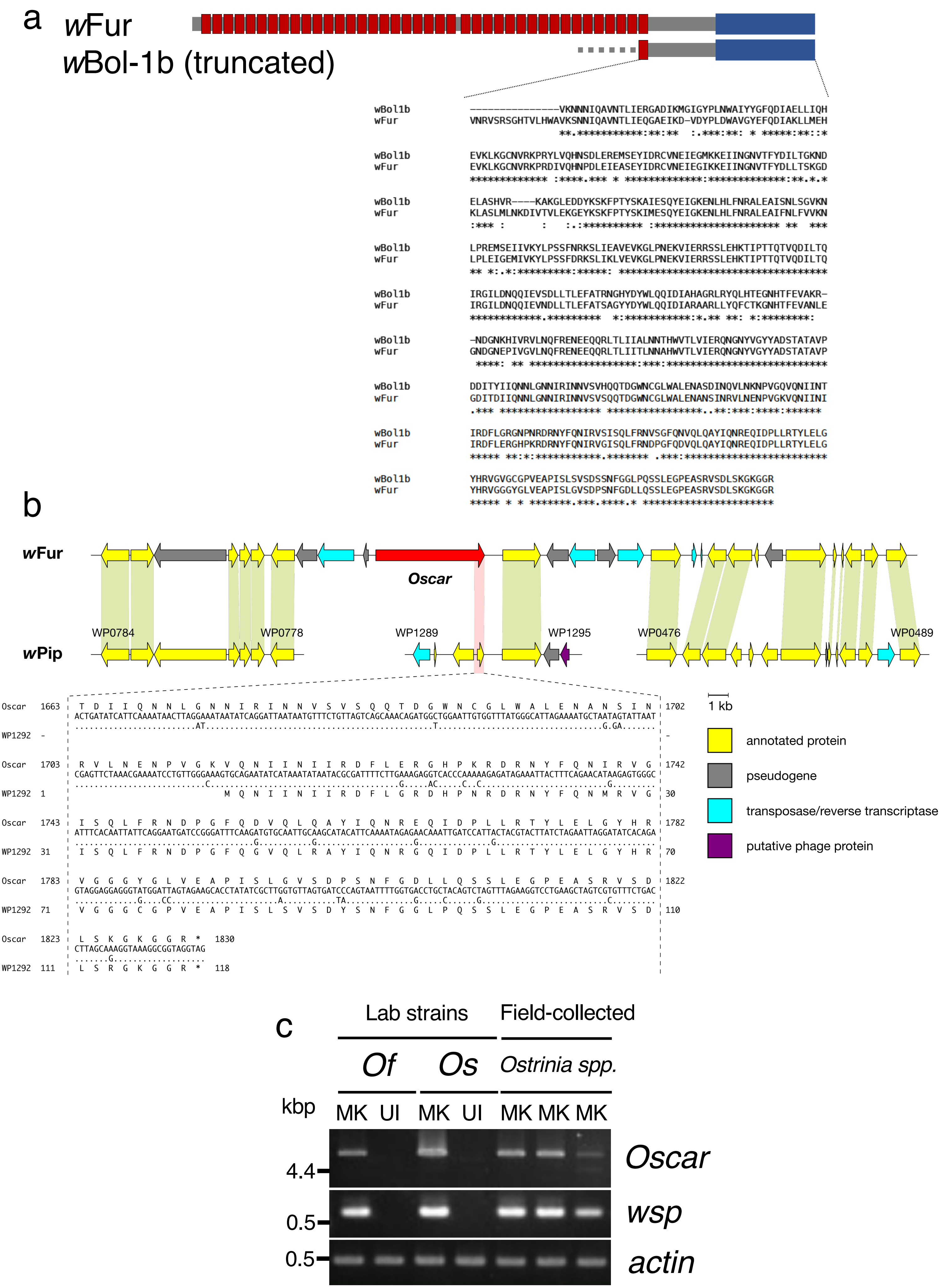

**Supplementary Fig. 6. Structures of *Oscar* gene and Oscar protein.**

a. Predicted structure of an Oscar homolog identified from the *w*Bol-1b genome. Alignment of amino acid sequences are also shown.

b. Synteny of the genomic regions around the *Oscar* locus of *w*Fur and *w*Pip. Nucleotide and amino acid alignments of *Oscar* (*w*Fur) and *WP1292* (*w*Pip) are also shown.

c. Detection of *Oscar* in *Ostrinia* female moths. *actin* and *wsp* were used as controls. Abbreviations: *Of*, *O. furnacalis*; *Os*, *O. scapularis*; MK, male-killing *Wolbachia*-infected; UI, uninfected. Similar results were obtained in two independent experiments of lab strains. Field-collected MK individuals (n = 3) were also used for this experiment.

Source data are provided as a Source Data file.

Supplementary Fig. 7

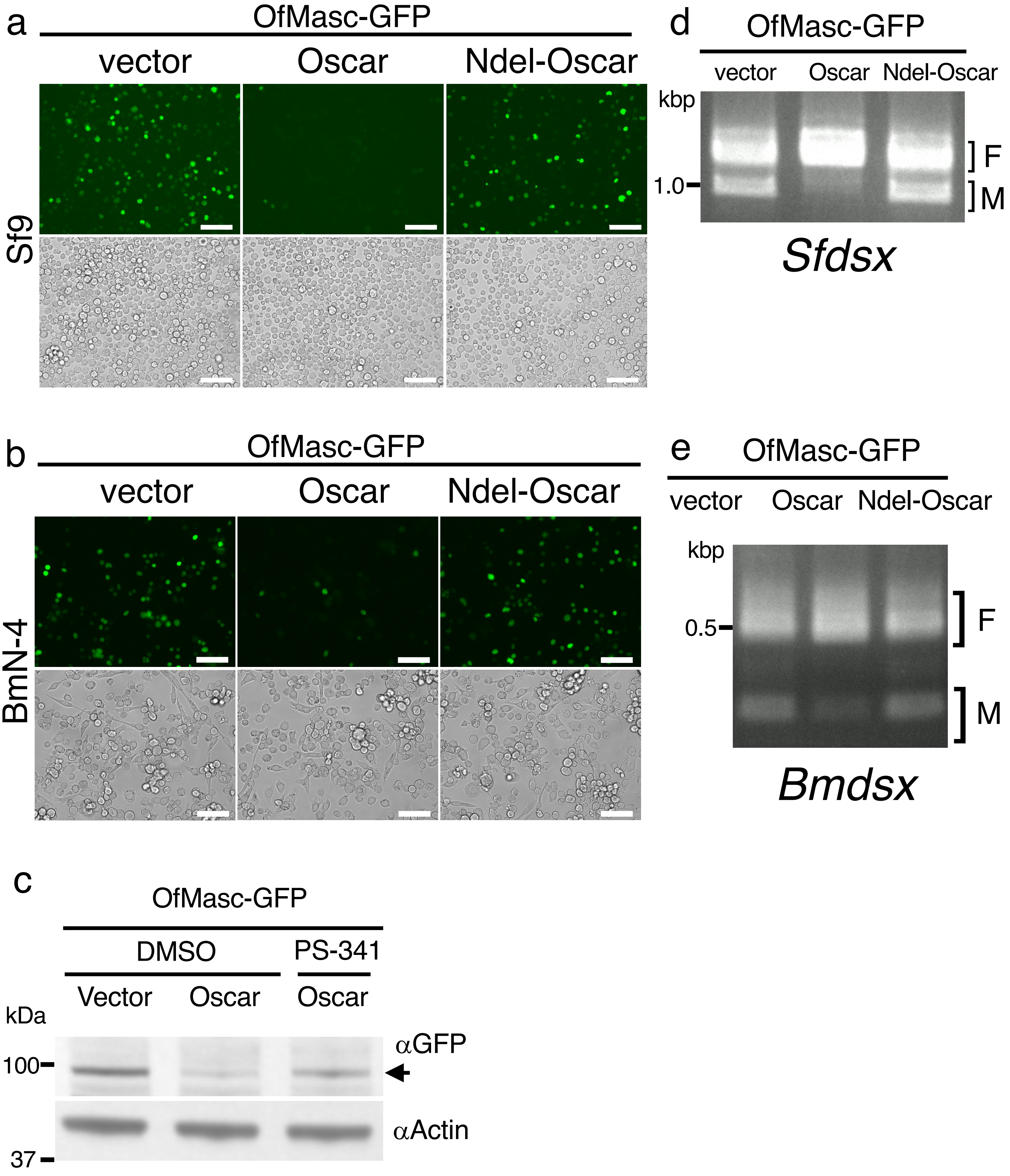

**Supplementary Fig. 7. Characterization of Oscar functions in lepidopteran cells.**

a. OfMasc accumulation in Oscar- or Ndel-Oscar-expressed Sf-9 cells. Bar, 100 µm. Similar results were obtained in two independent experiments.

b. OfMasc accumulation in Oscar- or Ndel-Oscar-expressed BmN-4 cells. Bar, 100 µm. Similar results were obtained in two independent experiments.

c. Immunoblots of OfMasc-GFP in Oscar-expressed OfT1C/tet cells treated with DMSO or PS-341. Cells were co-transfected with *OfMasc-GFP* and an empty vector or *Oscar* cDNA, treated with DMSO or PS-341 (200 nM) at 2 days after transfection, and harvested for immunoblotting at 3 days after transfection. Actin was used as a control. Similar results were obtained in two independent experiments.

d. Splicing patterns of *S. frugiperda dsx* (*Sfdsx*). Sf-9 cells were co-transfected with *OfMasc-GFP* and an empty vector, *Oscar* or *Ndel-Oscar* cDNA. Subsequently, *Sfdsx* splicing was investigated. The F and M indicate female- and male-type splicing of *Sfdsx*, respectively. Similar results were obtained in two independent experiments.

e. Splicing patterns of *B. mori dsx* (*Bmdsx*). BmN-4 cells were co-transfected with *OfMasc-GFP* and an empty vector, *Oscar*, or *Ndel-Oscar* cDNA. Subsequently, *Bmdsx* splicing was investigated. The F and M indicate female- and male-type splicing of *Bmdsx*, respectively. Similar results were obtained in two independent experiments.

Source data are provided as a Source Data file.

Supplementary Fig. 8

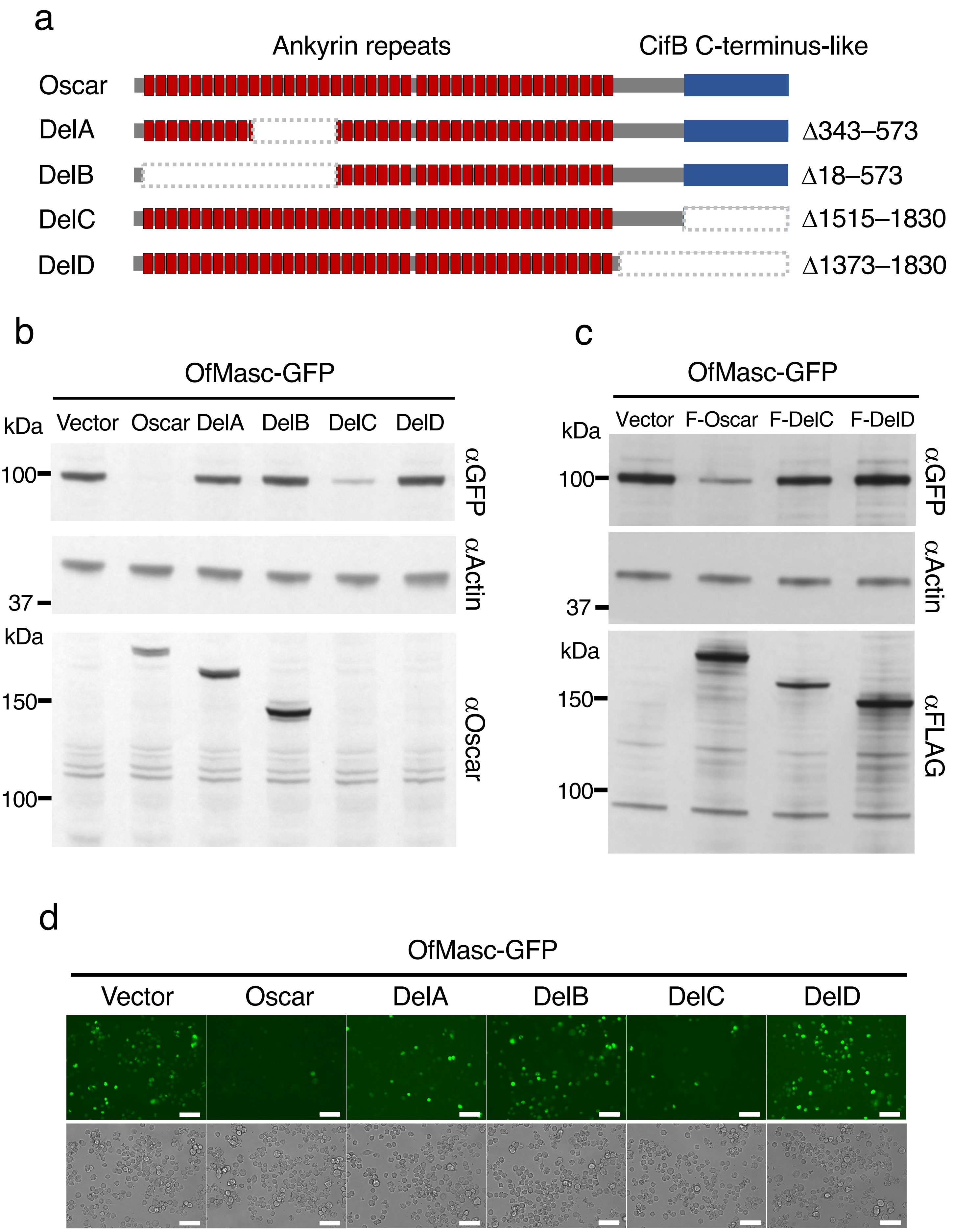

e

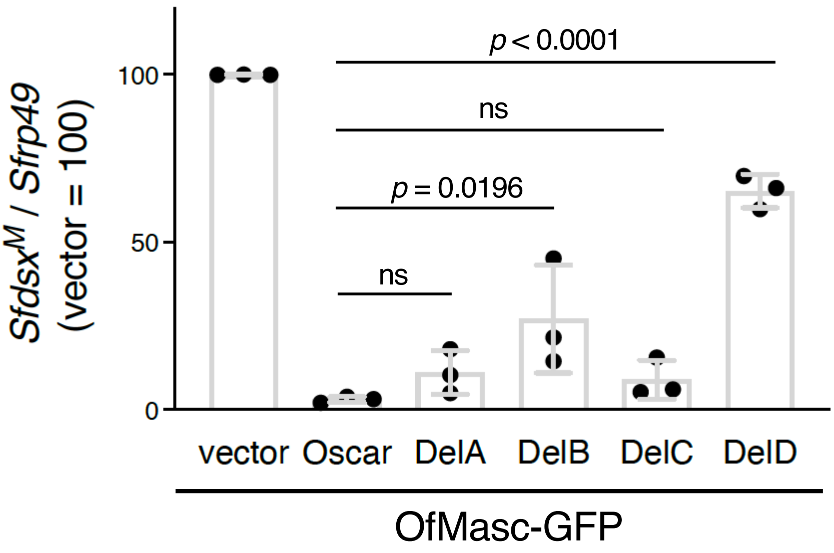


f

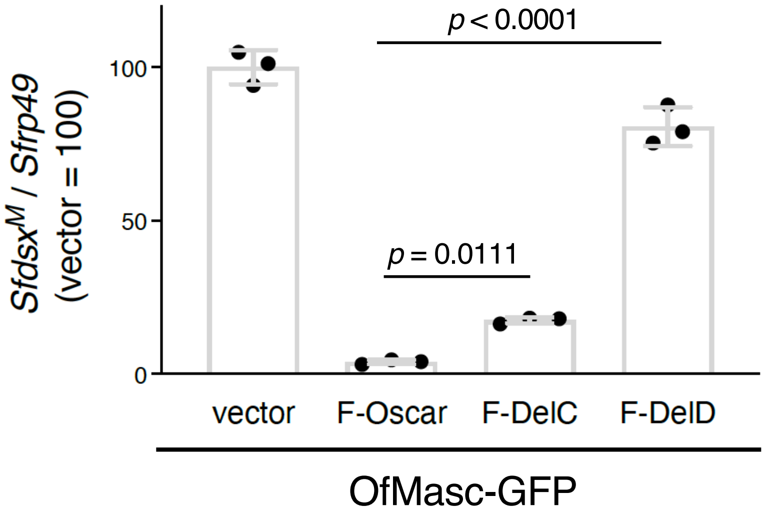


**Supplementary Fig. 8. Identification of functional regions essential for the Oscar function.**

a. Structure of Oscar derivatives.

b. Immunoblots of OfMasc-GFP and Oscar derivatives in Sf-9 cells. Actin was used as a control. Similar results were obtained in two independent experiments.

c. Immunoblots of OfMasc-GFP and FLAG-tagged Oscar derivatives in Sf-9 cells. Actin was used as a control. Similar results were obtained in two independent experiments.

d. Fluorescence microscopy of Sf-9 cells transfected with *OfMasc-GFP* and empty vector, *Oscar*, *DelA*, *DelB*, *DelC* or *DelD* cDNAs. Bar, 100 µm. Similar results were obtained in two independent experiments.

e. Quantification of the male-type splice variants of S*fdsx* (*Sfdsx*$^{M}$) in Sf9 cells transfected with *Oscar* derivatives. *Sfdsx*$^{M}$ levels were estimated by RT-qPCR. Data shown are means ± SD of three independent experiments. Adjusted $p$ values following one-way ANOVA with Dunnett's multiple comparisons tests are shown. ns, $p > 0.05$.

f. Quantification of *Sfdsx*$^{M}$ in Sf9 cells transfected with *3×FLAG-tagged Oscar* derivatives. *Sfdsx*$^{M}$ levels were estimated by RT-qPCR. Data shown are means ± SD in triplicate. Similar results were obtained in two independent experiments. Adjusted $p$ values following one-way ANOVA with Dunnett's multiple comparisons tests are shown.

Source data are provided as a Source Data file.

Supplementary Fig. 9

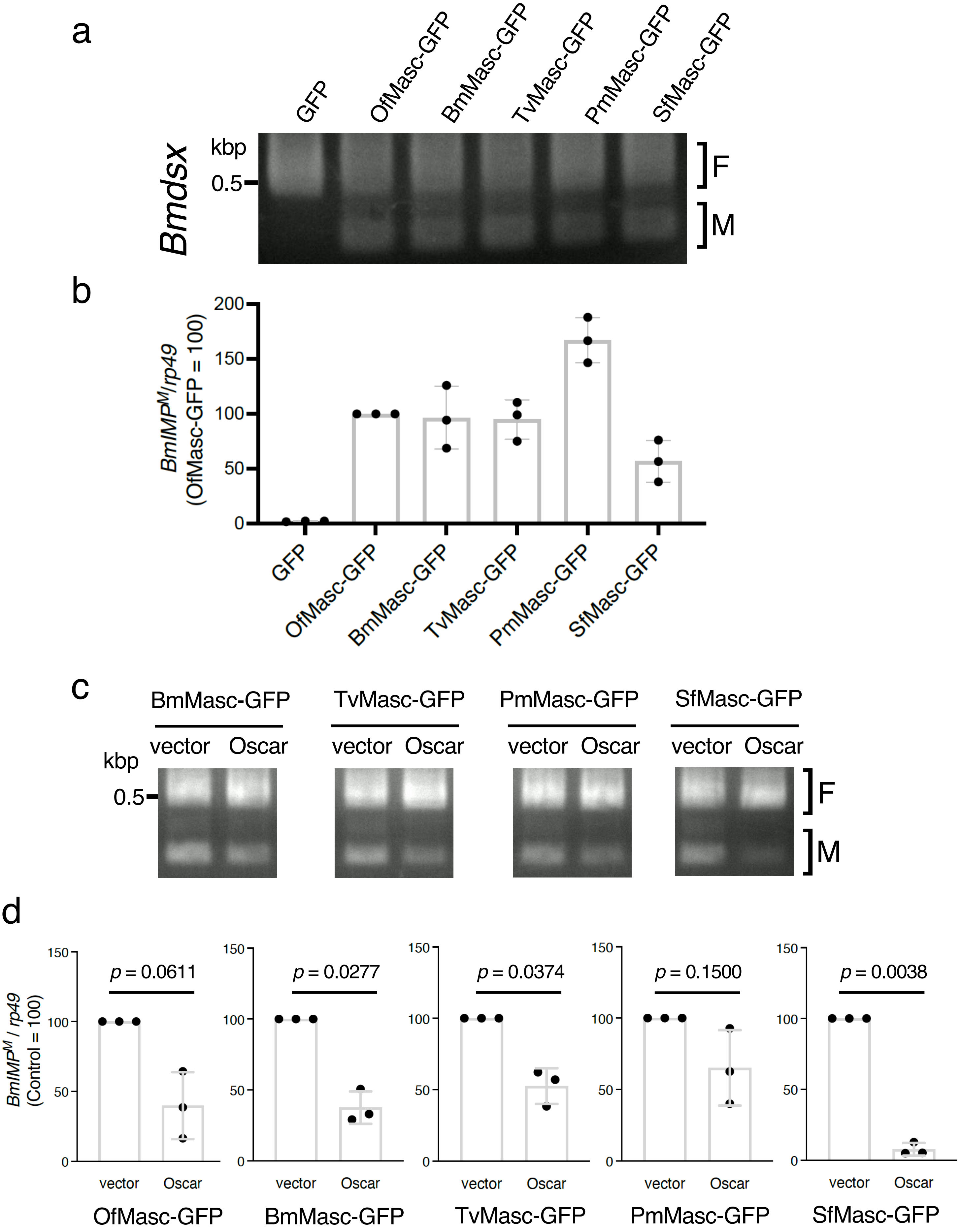

**Supplementary Fig. 9. Oscar inhibits lepidopteran Masc-induced masuculinization in BmN-4 cells.**

a. Splicing patterns of *Bmdsx* in Masc-transfected cells. BmN-4 cells were transfected with *GFP*, *OfMasc-GFP*, *BmMasc-GFP*, *Trilocha varians Masc* (*TvMasc*)*-GFP*, *Papilio machaon Masc* (*PmMasc*)-GFP or *S. frugiperda Masc* (*SfMasc*)*-GFP* cDNA, and *Bmdsx* splicing was investigated. The F and M indicate female- and male-type splicing of *Bmdsx*, respectively. Similar results were obtained in two independent experiments.

b. Expression of *BmIMP*$^M$ in Masc-transfected cells. BmN-4 cells were transfected with *GFP*, *OfMasc-GFP*, *BmMasc-GFP*, *TvMasc-GFP*, *PmMasc*-GFP or *SfMasc-GFP* cDNA, and the mRNA levels of *BmIMP*$^M$ were examined by RT-qPCR. The *BmIMP*$^M$ mRNA level was normalized to that of *rp49*. The data are shown as means ± SD of three independent experiments.

c. Effect of Oscar on *Bmdsx* splicing. BmN-4 cells were co-transfected with *Masc-GFP* and an empty vector or *Oscar* cDNA, and subsequently, *Bmdsx* splicing was investigated. The F and M indicate female- and male-type splicing of *Bmdsx*, respectively. Similar results were obtained in two independent experiments.

d. Effect of Oscar on *BmIMP*$^M$ expression. BmN-4 cells were co-transfected with *Masc-GFP* and an empty vector or *Oscar*, and subsequently, the mRNA levels of *BmIMP*$^M$ were examined by RT-qPCR. The *BmIMP*$^M$ mRNA level was normalized to that of *rp49*. The data are shown as means ± SD of three independent experiments. $p$ values were estimated by one sample $t$ test (two-tailed) with Benjamini-Hochberg correction.

Source data are provided as a Source Data file.

Supplementary Fig. 10

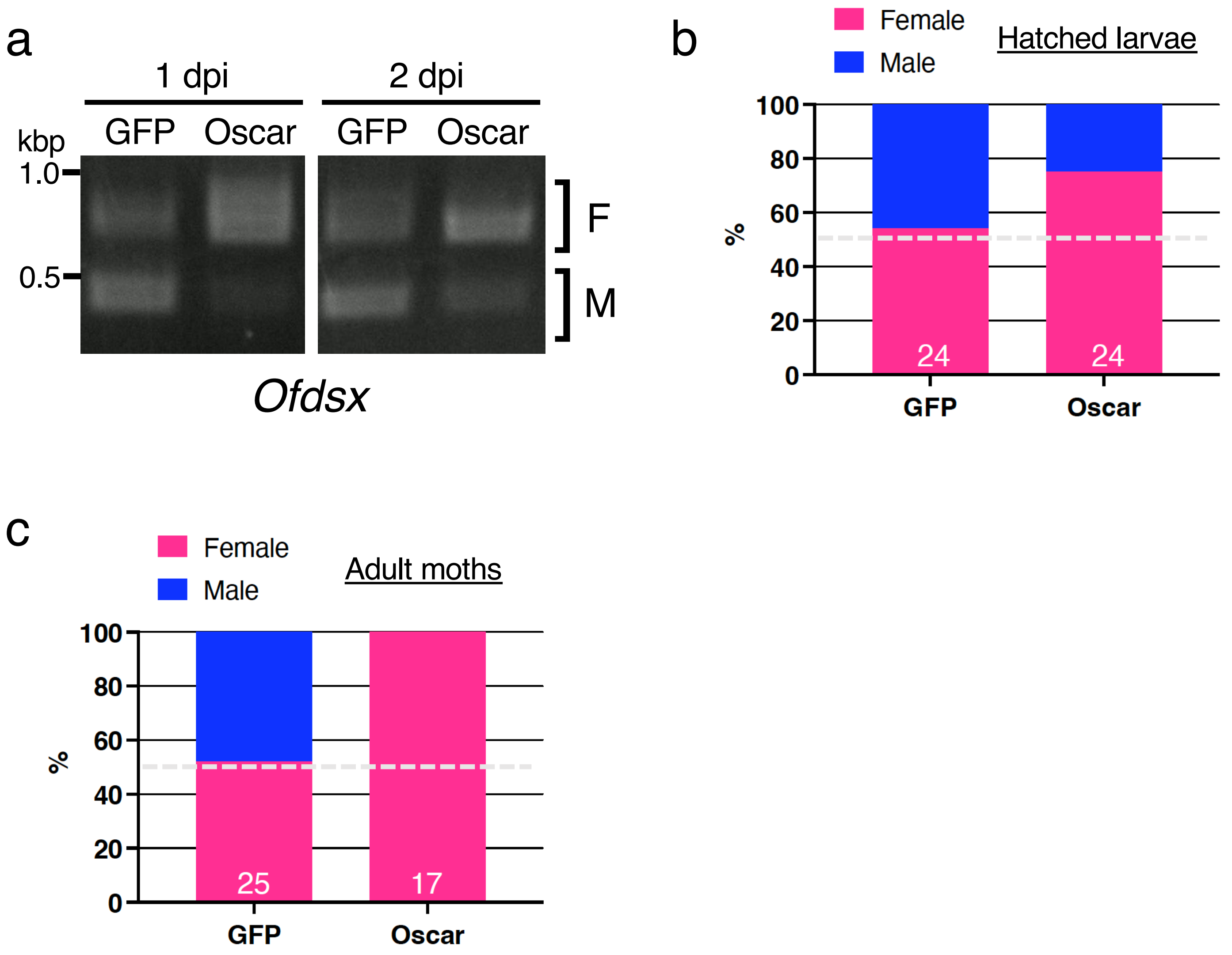


**<u>Supplementary Fig. 10. *Oscar* cRNA injection inhibits OfMasc-induced masculinization and leads to female-biased progeny production in *O. furnacalis*.</u>**

a. Splicing patterns of *Ofdsx* in *GFP* or *Oscar* cRNA-injected *O. furnacalis* embryos. At 1 or 2 days post-injection (dpi), total RNA was prepared from *O. furnacalis* egg masses, each of which contained more than 50 eggs that were all injected with *GFP* or *Oscar* cRNA. The F and M indicate female- and male-type splicing of *Ofdsx*, respectively. Similar results were obtained in two independent experiments.

b. *GFP* or *Oscar* cRNA was injected into *O. furnacalis* embryos, and the hatched larvae were collected and molecularly sexed. The number indicates the sample size of each group (*GFP*, n = 24; *Oscar*, n = 24).

c. The adult *O. furnacalis* moths emerged from *GFP* or *Oscar* cRNA-injected embryos were sexed based on the morphology. The number indicates the sample size of each group (*GFP*, n = 25; *Oscar*, n = 17).

Supplementary Fig. 11

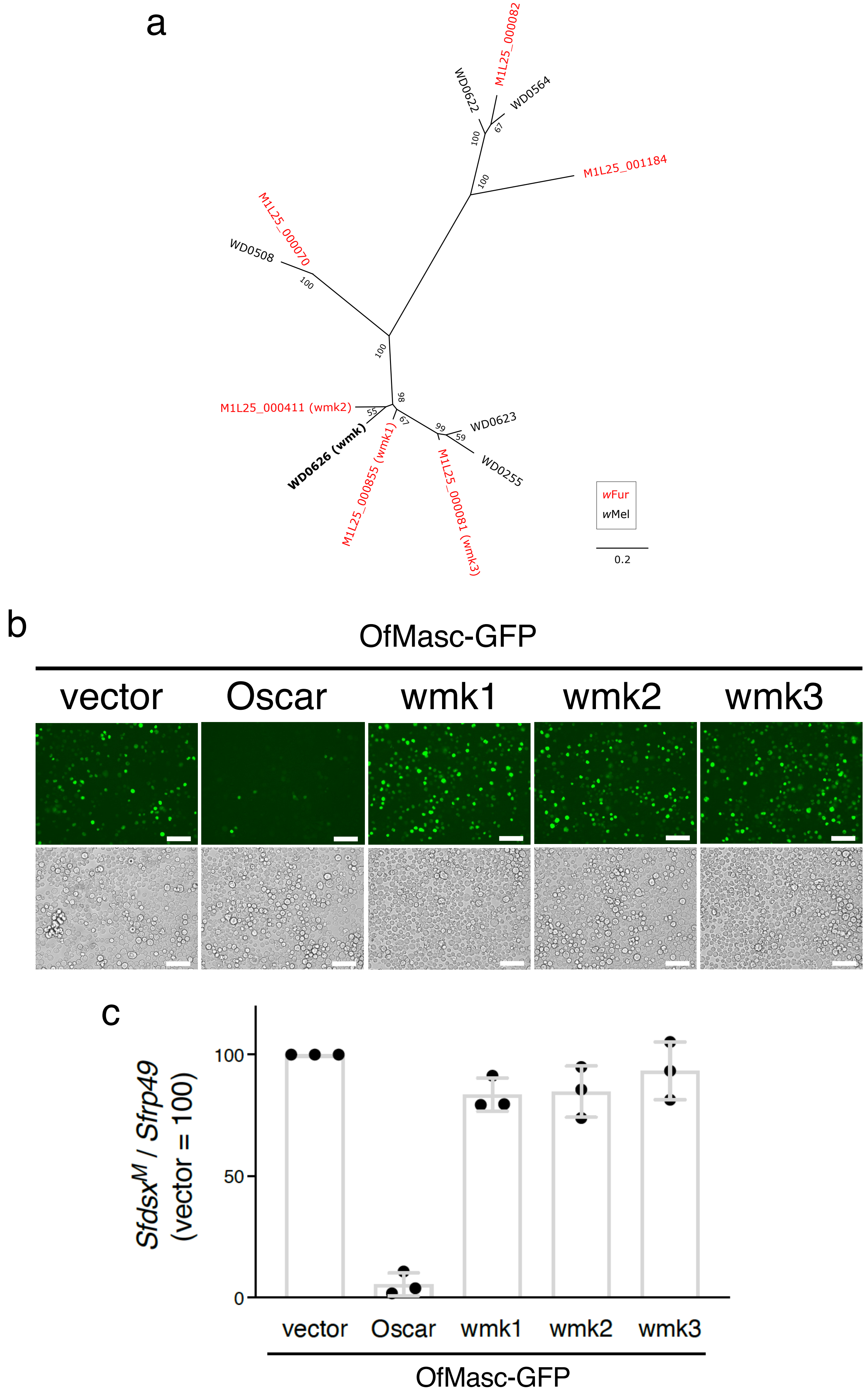

**Supplementary Fig. 11. Characterization of *w*Fur *wmk* and *wmk-like* genes.**
a. An unrooted tree of *w*Fur and *w*Mel Wmk homologs. The maximum likelihood tree was constructed based on amino acid sequences. Branch support calculated using 1000 bootstrap replicates is shown on the nodes. *w*Fur homologs are shown in red.
b. Fluorescence microscopy of Sf-9 cells transfected with *OfMasc-GFP* and empty vector, *Oscar*, *wmk1*, *wmk2* or *wmk3* cDNAs. Bar, 100 µm. Similar results were obtained in two independent experiments.
c. Quantification of the male-type splice variants of S*fdsx* (*Sfdsx*$^{M}$). *Sfdsx*$^{M}$ levels were estimated by RT-qPCR. Data shown are means ± SD of three independent experiments.
Source data are provided as a Source Data file.
