# Supplementary material for: A Wolbachia factor for male killing in lepidopteran insects
Source: Nat Commun. 2022 Nov 14;13:6764. doi: 10.1038/s41467-022-34488-y (PMC9663696; doi:10.1038/s41467-022-34488-y)
Supplement: Supplementary file 3 — Description of Additional Supplementary Files [file 41467_2022_34488_MOESM3_ESM.pdf]

Description of Additional Supplementary Files:

Supplementary Data 1: Nucleotide sequences of codon-optimized genes

Supplementary Data 2: Raw data of Fig. 2b (LC-MS/MS analysis)
