# Supplementary material for: A Wolbachia factor for male killing in lepidopteran insects
Source: Nat Commun. 2022 Nov 14;13:6764. doi: 10.1038/s41467-022-34488-y (PMC9663696; doi:10.1038/s41467-022-34488-y)
Supplement: Supplementary file 4 — Supplementary Data 1 [file 41467_2022_34488_MOESM4_ESM.zip › suppdata1.docx]

**Supplementary Data 1. Sequences of codon-optimized genes.**

>PmMasc (original)

ATGAACAATCACAGTGATCAAAATGGCCGCCAGCCCCCGCTGCCCGCGCAGCCACGGCCCCCTGCACGGACTGAAACAGAAAATACGACATCAAGCAATGAACCTAAGCAAATATGTCGTAACTATGTCTGGGGCACATGCAAGAAAAATACGAAATGTAAATTTCGTCACGAACTGGATTTTGAAGAAATGAAAAAAATTCTTAAATTCTGTCACGACTATCAAAACAAAACTGGCTGCACGAGGGGAGACTGTACATACCTACATACGACTAGAGAAGAGGAAAATCTTTTTCTGGCTACAGGGGAAATACCTCGTGTGCTTGCAGAACGTCATGCCGCCATGCAAAAAGCGGCAACGGCGAACAATGGCGAGACAATACGGCAGATAGCAGTGTTTATTAGAGAATCTTATGTACCTCCCCAACAGAATGGATTGCCGGGGCCCATGCCGCCTTCGTCGGTACCCCCCGGCGCGACAGGCTGTGCAGCGAGGCCGGCGCCGCCGCCGCCTCCACCATCAGTCCATGCGATGTCGGTGATGCCAGCCATGCCGCTTCAAATCATGCCACTGCCAGTCCAGCAACCACCGCCTCCTCCGCCTCCCCCACCGCCCCCGCCGCCACCTCCTACAGCTTCCCCGAGTGATCCACGGCCCACAGCCCCTGTTTATACAGCACCACTTGGTCAACCTCCAGAAAGAACAACAGTGGTTTTTCCAATGAATCAACCACCTCCTCCTCTGCCTGTCATGTTTGACGCAAGTAAACCTCCACCACCATTACCCAATCAAATAAAGGCGAGTAGTGTTGCTGTGAAGCATAAAGCAGTAAGTTTACCTGAAGCTGGACCAAGCAAAATAAGGAAGCCAGGGGACAACAATGTAGCAGACGCGCATTGCGAAAACTGTGTTCAACGCAAGATCAGGATCGATTTATACTGCCAAGAATTAGATAAACTACAATGTGAACAGGAATATCAAAAATTGCTCTATGAAAAGAACCTGGAAGAGTACAAAACTGCAAAAGAACTTTTGAGATCTGTAGTCAGTGCTGACTTGTTTCGGATTTTGGAAGAACATATCGAGGGAGTACCACAAATACAAGATATTATGTCTCACTTAAACCCAACGTCATTTGGTTCGAGTATGTCCACAGTACCAAAACAATTTTTGTTACAACTTATGGATTGTGTTTTAAGTAATCCAAGGAATGTACTAGATATGGGTTCCAGTACTTCAAGCAATTTGGATGAAAGTTTTCTGCCATCTTTATCATCCGTGTCTAGAAGGACAAATTCAATTTTAAATCATTCATCACCTACAGATGTTATACAAGCCTTTGTTGATCTGCTTCAGCATTCTAAAATAAGTCCAGATAAACAACAATCATCTGACTTTTCAAATGAAATAAACAGATTAGCTGGTACAAATGGCAGTAACGGCATGAATGGTATCACCGGTTGCAGTACTAGTCAGAAAGTGGAAAGTTCAACTGCAACATCAATGGCGGGATCAATTACACATAGCGGGAGCGTGAGCATGTCCTCTTTTGCTGGGGCACTAATGACTGTCCCTCCTCCACCTTTCCCTTCTTACCAACCAACCATCCCTCCACCACCACCCACTGGGTCGTACTATAACTAG

>PmMasc (codon-optimized)

ATGAACAACCACTCGGATCAGAACGGACGACAACCACCTTTGCCAGCCCAGCCTCGACCTCCGGCACGAACTGAAACAGAGAATACCACCAGTTCAAATGAGCCAAAGCAGATATGCAGGAATTACGTCTGGGGCACGTGTAAGAAGAACACAAAGTGCAAGTTCAGGCACGAGTTAGATTTCGAGGAAATGAAGAAGATTCTTAAATTCTGTCATGATTATCAAAACAAAACGGGTTGTACGCGAGGTGATTGTACGTACTTGCACACCACCCGTGAAGAAGAAAACTTATTCCTCGCAACCGGCGAAATTCCGAGAGTATTAGCAGAGCGCCATGCCGCAATGCAGAAAGCAGCGACGGCTAATAACGGTGAAACAATACGTCAGATCGCAGTGTTCATTCGCGAATCGTATGTACCTCCACAACAGAACGGTCTCCCTGGACCAATGCCGCCGAGTTCTGTACCGCCTGGTGCAACGGGCTGCGCTGCTAGGCCAGCCCCGCCTCCTCCACCACCTTCAGTCCATGCCATGTCTGTAATGCCAGCAATGCCCCTCCAGATCATGCCTCTGCCCGTCCAACAGCCGCCGCCGCCACCTCCACCACCACCTCCGCCGCCTCCACCACCAACGGCTTCCCCTTCAGATCCTAGACCGACTGCCCCAGTGTACACAGCACCATTAGGACAGCCACCCGAGAGGACAACGGTCGTCTTCCCTATGAACCAACCACCACCGCCATTGCCGGTCATGTTCGACGCATCAAAGCCTCCTCCTCCGTTGCCTAACCAGATCAAGGCCAGTTCCGTAGCAGTAAAGCATAAGGCAGTCTCTCTGCCCGAAGCGGGACCCTCTAAAATAAGAAAGCCAGGAGATAACAATGTGGCGGATGCTCACTGTGAGAACTGCGTTCAGCGAAAGATCCGTATTGACTTGTACTGCCAAGAACTCGACAAGCTCCAGTGTGAACAAGAGTACCAAAAGTTGCTTTACGAGAAGAACTTGGAGGAATATAAGACTGCAAAGGAGCTGCTGCGCAGTGTGGTTTCTGCCGATCTATTCCGCATCTTAGAGGAGCACATAGAGGGAGTCCCGCAAATACAAGACATTATGAGTCACCTCAACCCGACGTCATTCGGTTCGTCTATGTCCACTGTACCTAAGCAATTCTTGTTGCAACTGATGGACTGCGTACTGTCCAATCCACGAAACGTTCTTGACATGGGCAGCTCAACATCTAGCAACCTTGATGAAAGCTTCCTTCCCTCATTATCTAGTGTTTCTCGTAGGACAAATTCTATTTTAAACCATTCAAGTCCGACTGACGTCATTCAGGCTTTCGTAGATCTTCTCCAGCACAGCAAGATCAGCCCAGACAAGCAACAGAGTTCCGACTTCTCCAATGAGATAAACCGCTTGGCTGGCACAAATGGCTCCAATGGCATGAATGGCATTACGGGTTGCAGTACTTCCCAGAAAGTTGAGAGCTCGACCGCGACGAGCATGGCAGGATCTATTACGCATTCAGGCTCCGTTAGTATGAGTTCATTTGCCGGGGCATTAATGACGGTTCCGCCTCCGCCATTCCCAAGTTATCAGCCTACAATTCCGCCACCTCCGCCCACCGGAAGTTACTACAACTAA

>Oscar (original)

ATGGAAGATAGACACATTCCTTTTCTTTCATCCTTAGCTATTTTGCTATGGTATGGTATTAATAAAGCAGATAGATATGGATATACTCCTTTACACTTAGCTGTTCAATCAGGTAATCTTGAGATAGTAAATGCTTTAGTAGGAAGAGGTGCTGATGTTGATAAAGCAGATAGATATGAACGTACTCCTTTACACTTAGCTGTTCAATCAGGTAATCTTGAGATAATAAATGCTTTAGTAGGAAGAGGTGCTGATGTTGATAAAGCAGATAGATATGAACGTACTCCTTTACACTTAGCTGTTCAATCAGGTAATCTTGAGATAATAAATGCTTTAGTAGGAAGAGGTGCTGATGTTAATAAAGCAGATAAGTATGGATCTACTCCTTTAAATTTTGCTGCTCAAATAGGTAATGCTGAGATAATAAATGCTCTAGTAGCACATGAAGTGGGTGATGATATATGTACACCTTTACATTTAGCTGTTCTAAGGAACGATATGGAGTTAGTAAATAGTCTAATAAAACAAGGGGCTAATGTTTGTCAAGAGAGTAGGGGCAGATATATACCTTTACATTTAGCTGCTCGAGCAGGTAATGTTGAGATGATAAATGCTCTAATAGAACAAGGTGCCAAGATTAATCAAATGAATAATGATAGATTTACTCCTTTACATTTTGCTGTACAAGTAGGTGATGTTGATGTAGTAAATGCTTTATTAGCAAGAAGTGCTAATGTTAATGAAGTAGGTAAGTATGGATTTACTCCTTTGCATCTTGCTACTCAAGTAAGCAATGCAGAGGTGATAAGAGCTCTGATAGAAAGAGGGGCTGATGTTAATAAAGCAGATAAATATGGATATACTCCTTTACACTTAGCTGTTCAATCAGGTAATCTTGAGATAATAAATGCTTTAGTAGGAAGAGGTGCTGATATTAATAAAGCAGATAAATATGAACGTGCTCCTTTACACTTAGCTGTACAAGTAGGTGATGTTAATGTAGTAAATGCTTTATTAGCAAGAAGTGCTAATGTTAATAAAGTAGATAGATATGGACATACTCCTTTACACTTAGCTGTTCAATCAGGTAATCTTGAGATAATAAATGCTTTAGTAGGAAGAGGTGCTGATATTAATAAAGCAGATAAATATGAACGTGCTCCTTTACACTTAGCTGTACAAGTAGGTGATGTTAATGTAGTAAATGCTTTATTAGCAAGAAGTGCTAATGTTAATAAAGTAGATAGATATGGACATACTCCTTTACACTTAGCTGTTCAATCAGGTAATCTTGAGATAATAAATGCTTTAGTAGGAAGAGGTGCTGATATTAATAAAGCAGATAAATATGAACGTGCTCCTTTACACTTAGCTGTACAAGTAGGTGATGTTAATGTAGTAAATGCTTTATTAGCAAGAAGTGCTAATGTTAATAAAGTAGATAGATATGGACATACTCCTTTACACTTAGCTGTTCAATCAGGTAATCTTGAGATAATAAATGCTTTAGTAGGAAGAGGTGCTGATATTAATAAAGCAGATAAATATGAACGTGCTCCTTTACACTTAGCTGTACAAGTAGGTGATGTTAATGTAGTAAATGCTTTAGTAGGAAGAGGTGCTGATATTAATAAAGCAGATAAATATGAACGTGCTCCTTTACACTTAGCTGTACAAGTAGGTGATGTTAATGTAGTAAATGCTTTATTAGCAAGAAGTGCTAATGTTAATAAAGTAGATAGATATGGACATACTCCTTTACACTTAGCTGTTCAATCAGGTAATCTTGAGATAATAAATGCTTTAGTAGGAAGAGGTGCTGATGTTAATAAAGCAGATAAGTATGGATCTACTCCTTTAAATTTTGCTGCTCAAATAGGTAATGCTGAGATAATAAATGCTCTAGTAGCACATGAAGTGGGTGATGATATATGTACACCTTTACATTTAGCTGTTCTAAGGAACGATATGGAGTTAGTAAATAGTCTAATAAAACAAGGGGCTAATGTTTGTCAAGAGAGTAGGGGCAGATATATACCTTTACATTTAGCTGCTCGAGCAGGTAATGTTGAGATGATAAATGCTCTAATAGAACAAGGTGCCAAGATTAATCAAATGAATAATGATGGATTTACTCCTTTACATTTTGCTGTACAAGTAGGTGATGTTGATGTAGTAAATGCTTTATTAGCAAGAAGTGCTAATGTTAATGAAGTAGGTAAGTATGGATTTACTCCTTTGCATTTTGCTGCTCAAGTAAGCAATGCAGAGGTGATAAGAGCTCTGATAGAAAGAGGGGCTGGCGTTAATCAAATGAATAGTGACGAATCTGTTTTCGGAAGTAGTGACAGATATGCTTACGGAAGCACTCCTTTACATTTAGCTATTGAGAAAGGCAATTTCGAGGCAGTGAATTGTTTATTAGAAGAAGGTGCTGATATTAATCAAACGGATGGATATGGACGTACTCCTTTACATTTAGCTATTGAGGAAGGCAATTTCGAGGCAGTGAATGCTTTAGTAGGAAGAGGTGCTGATGTTAATAAAGCAGATAGATATGAACGTACTCCTTTATACTTAGCTGTTCAACTAGGTAATCTTGAGATGGTAAATGCTTTAGTAGGAAGAGGTGCTGATATTAATAAAGCAGATAAATATGAACGTACTCCTTTAAACTTAGCTGTTCAATTAGGTAATGCTGAGATAATAAATGCTCTAATAGCACATGAAGTGGGTGATGATATATGTACACCTTTACATTTAGCTGTTCTAAGAAACGATATGGAGTTAGTAAATAGTCTAATAGAACGAGGGGTTGATGTTAATAAAGCAAATGAGTATGGATTTACTCCTTTACATTTTGCTATTGAGAGAGGCAATTTCGATGCAGTGAATGCTCTAATATCACATCAAGTGGGTGATGATGATAGATACACACCTTTACATTTAGCTGTTCTAAGGAAGAACGATATAGGGTTAGTAAATAGTCTAATAGAACAAGGGGCTGATGTTGATGAAGTAAGCAGAGATAGATACACTCCTTTACATTTAGCTGTTAGAAGCAACAATATACAGATGGTAGATACTCTAATAAAACGAGGTGCTAATGTTAATCGGATAAATAGGGATGGGTATACTCCTTTGCACTTAGCTGTTCAAGTAGGTAATGTTGAGCTGATCAATGCTCTAATAAAAGGAAGTGCCAATATTAATAAGGTGGATAGACATGGGCATACTGTTTTGCATTTGGCTATTAAAAGCAACAATATACAGGTAGTAAATGCCCTAATAGAACGGGGGGTTGATGTTAATAAAGCAAATAGGTATGGATTTACTCCTTTACATTTTGCTGTTAGAAGCAACAATATACAGATGGTAGATACTCTAATAAAACGAGGTGCTAATGTTAATCGGATAAATAGGGATGGGTATACTCCTTTGCACTTAGCTGTTCAAGTAGGTAATGTTGAGCTGATCAATGCTCTAATAAAAGGAAGTGCCAATATTAATAAGGTGGATAGACATGGGCATACTGTTTTGCATTTGGCTATTAAAAGCAACAATATACAGGTAGTAAATGCCCTAACAGAACGGGGGGCTGATGTTAATAAAGCAGATAGGTATGGATTTACTCCTTTACATTTTGCTGTTGAAAGAGGCAATTTTGAGGTAGTAGATACTCTAATAAAACGAGGGGCTGATGTTAATAGGGTGGGTAGACGTGGACATACTGCTTTACATTGGGCTATCAGGGAGAACAATATACAGGTAGTAAATGCTCTAATAGAACGAGGGGCTGATGTTAATAGGGTGGGTAGACGTGGACATACTGCTTTACATTGGGCTATCAGGGAGAACAATATACAGGTAGTAAATGCTCTAATAGAACGAGGGGCTGATGTTAATAGGGTGAGTAGAAGTGGACATACTGTCTTACATTGGGCTGTTAAAAGTAATAATATACAGGCAGTAAATACCCTAATAGAACAAGGAGCTGAAATTAAGGATGTTGACTATCCTTTAGATTGGGCTGTAGGTTATGAGTTTCAAGATATAGCAAAGCTTTTAATGGAACATGAAGTTAAGTTAAAGGGTTGCAATGTAAGAAAGCCACGAGATATCGTGCAACATAATCCCGATCTTGAAATAGAAGCGTCAGAATATATTGATAGATGTGTGAATGAAATAGAAGGGATAAAAAAAGAAATAATTAATGGTAATGTTACATTTTACGATCTTTTGACAAGCAAAGGTGATAAATTAGCATCTTTGATGCTTAATAAAGATATAGTAACAGTATTGGAGAAAGGTGAATATAAAAGTAAATTTCCTACGTATTCCAAGATAATGGAAAGTCAATATGAAATAGGAAAGGAGAATTTACACCTATTCAATCGAGCTTTGGAAGCAATCTTCAATCTTTTTGTAGTGAAAAATTTACCTCTAGAAATAGGTGAAATGATAGTAAAATATCTACCAAGTAGTTTCGATAGAAAATCCTTAATTAAGTTGGTAGAGGTGAAAGGGCTACCTAATGAGAAAGTGATAGAGAGAAGGTCTTCATTAGAGCATAAAACTATACCTACTACTCAAACTGTGCAAGACATATTAACACAAATAAGAGGGATTCTTGATAACCAACAGATAGAAGTAAATGATCTGCTTACACTGGAATTTGCAACAAGTGCTGGCTATTATGATTACTGGCTTCAACAAATTGATATAGCCCGTGCTGCAAGATTGCTATATCAGTTTTGTACTAAAGGTAATCACACTTTTGAAGTAGCTAATTTGGAGGGGAATGACGGTAACGAGCCCATTGTTGGAGTATTAAATCAATTTAGGGAAAATGAAGAACAACAGCGTTTAACCTTAATCATTACTTTAAATAATGCTCATTGGGTTACACTAGTGATTGAACGTCAAAATGGGAACTATGTTGGATACTATGCTGATTCAACTGCTACTGCTGTTCCAGGTGATATTACTGATATCATTCAAAATAACTTAGGAAATAATATCAGGATTAATAATGTTTCTGTTAGTCAGCAAACAGATGGCTGGAATTGTGGTTTATGGGCATTAGAAAATGCTAATAGTATTAATCGAGTTCTAAACGAAAATCCTGTTGGGAAAGTGCAGAATATCATAAATATAATACGCGATTTTCTTGAAAGAGGTCACCCAAAAAGAGATAGAAATTACTTTCAGAACATAAGAGTGGGCATTTCACAATTATTCAGGAATGATCCGGGATTTCAAGATGTGCAATTGCAAGCATACATTCAAAATAGAGAACAAATTGATCCATTACTACGTACTTATCTAGAATTAGGATATCACAGAGTAGGAGGAGGGTATGGATTAGTAGAAGCACCTATATCGCTTGGTGTTAGTGATCCCAGTAATTTTGGTGACCTGCTACAGTCTAGTTTAGAAGGTCCTGAAGCTAGTCGTGTTTCTGACCTTAGCAAAGGTAAAGGCGGTAGGTAG

>Oscar (codon-optimized)

ATGGAGGATAGACATATCCCGTTCCTGTCCTCATTAGCCATATTGTTATGGTATGGAATTAATAAAGCCGACCGCTATGGGTACACACCATTGCATCTCGCCGTTCAGTCGGGTAACCTCGAGATTGTCAACGCGCTGGTCGGACGTGGCGCTGATGTGGACAAGGCTGATAGATACGAGCGTACTCCGCTTCATCTCGCTGTCCAGTCCGGAAATCTGGAGATTATAAATGCCCTCGTAGGGCGTGGCGCTGATGTTGACAAAGCGGACCGGTACGAAAGAACACCCCTGCACCTGGCCGTCCAGTCTGGAAACCTAGAGATAATAAACGCACTTGTAGGTAGGGGGGCTGACGTGAACAAGGCAGACAAATATGGCTCAACTCCACTGAATTTCGCCGCACAGATTGGTAACGCCGAAATCATAAACGCTCTTGTGGCACACGAAGTCGGTGACGATATTTGCACGCCTCTTCATCTGGCAGTTTTGCGAAATGATATGGAACTGGTGAATTCGTTAATTAAACAGGGGGCCAATGTTTGCCAAGAATCGCGCGGTCGCTACATACCCTTGCATTTGGCTGCCCGAGCCGGCAACGTGGAAATGATCAACGCCCTCATTGAACAAGGCGCCAAGATAAACCAGATGAACAATGATCGCTTCACTCCATTACATTTCGCAGTCCAAGTAGGAGACGTCGATGTCGTGAATGCTTTATTGGCTCGATCAGCCAACGTAAACGAGGTCGGTAAATATGGATTTACACCACTCCACCTTGCAACTCAGGTTAGTAATGCGGAAGTGATACGTGCTCTGATAGAGAGGGGCGCAGATGTTAATAAGGCGGACAAGTATGGATACACCCCATTGCACCTAGCTGTCCAATCTGGTAACTTGGAAATCATCAATGCGCTTGTCGGACGAGGTGCTGACATCAACAAGGCAGACAAGTACGAGCGTGCGCCACTTCACCTCGCGGTGCAGGTGGGCGACGTAAATGTAGTGAATGCGCTATTAGCTCGCAGTGCCAATGTAAACAAGGTAGATCGTTACGGACACACGCCCTTACACTTGGCGGTTCAATCTGGAAATCTTGAAATTATCAACGCACTCGTTGGACGTGGTGCTGATATAAACAAAGCCGATAAGTACGAGAGAGCACCTCTACACCTAGCAGTGCAAGTCGGCGACGTAAACGTCGTGAACGCACTTCTGGCACGTAGTGCGAACGTTAATAAAGTAGATCGATATGGACACACGCCACTGCACTTAGCTGTGCAGTCAGGGAACTTAGAAATCATAAACGCGCTAGTAGGACGTGGAGCTGACATCAACAAAGCGGATAAATATGAACGCGCTCCTCTACATCTTGCTGTGCAGGTCGGGGATGTAAACGTGGTCAACGCTCTGTTAGCCCGCTCTGCTAACGTGAACAAGGTCGATAGATACGGTCACACCCCGCTTCACTTGGCCGTGCAAAGCGGCAACCTCGAAATTATTAACGCGCTGGTTGGCAGGGGAGCAGATATAAATAAAGCTGATAAATACGAAAGAGCTCCTTTGCACCTCGCTGTTCAGGTTGGAGACGTGAATGTGGTTAACGCTCTGGTAGGTCGAGGCGCTGACATAAACAAGGCCGACAAGTATGAAAGGGCTCCCTTACACCTCGCTGTACAAGTAGGTGATGTGAACGTTGTCAATGCTCTGCTTGCTCGTAGCGCTAACGTAAATAAAGTGGACCGTTACGGTCATACTCCGCTCCACCTGGCAGTTCAATCGGGCAACTTGGAGATCATTAATGCGTTGGTTGGAAGGGGCGCCGACGTGAATAAGGCCGACAAATACGGCAGTACTCCACTAAACTTTGCAGCGCAGATTGGGAACGCTGAAATAATCAATGCTTTGGTTGCGCATGAGGTAGGCGATGATATCTGTACCCCACTCCATTTAGCCGTGCTGAGGAACGACATGGAGCTCGTTAATTCGCTGATTAAACAAGGGGCAAACGTGTGTCAGGAGAGCCGGGGGCGCTACATCCCTCTCCACCTTGCTGCACGTGCCGGAAACGTAGAGATGATAAATGCTCTCATAGAGCAGGGTGCAAAGATCAATCAGATGAACAACGATGGTTTTACACCGTTGCATTTTGCGGTGCAAGTGGGTGACGTGGACGTCGTAAATGCATTACTGGCGAGATCGGCGAATGTGAATGAAGTCGGAAAATACGGTTTCACGCCCCTCCATTTTGCCGCTCAAGTCTCCAATGCTGAAGTGATAAGAGCACTGATAGAGCGTGGTGCCGGGGTTAATCAAATGAATTCAGATGAGAGTGTCTTTGGAAGCTCAGATCGATACGCCTACGGCTCTACGCCTTTACATCTAGCTATTGAAAAAGGCAATTTTGAAGCTGTTAATTGTTTATTGGAAGAAGGAGCTGATATCAATCAAACAGATGGTTATGGCCGAACCCCTTTACACCTGGCGATCGAGGAAGGCAATTTCGAAGCAGTTAATGCGCTGGTTGGTCGGGGCGCTGACGTTAATAAGGCAGACCGATACGAACGCACACCTCTCTACTTGGCGGTCCAACTCGGCAATTTGGAGATGGTGAACGCTCTAGTCGGCAGGGGAGCCGACATCAATAAGGCTGATAAATACGAACGGACTCCTTTGAACCTAGCGGTTCAGCTTGGCAACGCCGAGATCATTAACGCTTTGATTGCTCACGAAGTTGGTGATGACATCTGCACCCCGCTGCACCTTGCAGTCTTGCGCAACGACATGGAGCTGGTCAATAGCCTAATAGAACGCGGTGTTGACGTTAACAAGGCAAATGAGTACGGATTCACCCCTCTGCATTTCGCGATAGAGAGAGGTAACTTCGACGCGGTAAATGCTTTAATATCCCACCAAGTGGGAGACGACGACCGTTATACCCCTCTTCATCTGGCAGTGCTTAGAAAAAATGACATCGGGCTCGTCAACAGTCTGATTGAACAGGGGGCTGATGTAGACGAAGTCAGCAGAGATCGGTATACACCGCTACATCTTGCAGTAAGATCGAACAATATACAGATGGTCGATACTTTAATAAAACGCGGAGCTAATGTTAACAGAATCAACCGAGACGGATACACACCCCTTCACTTAGCCGTCCAAGTGGGAAATGTGGAACTCATAAATGCGCTAATTAAAGGTTCGGCTAACATTAATAAGGTTGATCGGCACGGCCATACTGTGTTACACCTCGCTATCAAAAGCAACAATATACAGGTTGTGAACGCATTAATTGAAAGGGGTGTAGATGTCAATAAAGCCAATCGCTATGGATTCACACCTTTACACTTTGCTGTGCGGTCCAATAATATTCAGATGGTAGACACTCTTATTAAGAGAGGCGCAAACGTGAATAGAATAAACCGGGACGGGTACACGCCCCTGCATCTAGCTGTCCAGGTTGGTAATGTTGAACTCATCAACGCATTGATCAAGGGATCTGCTAATATTAACAAAGTTGATCGACATGGTCACACTGTACTCCACTTAGCGATAAAGTCCAACAATATTCAAGTTGTCAACGCTTTGACCGAAAGAGGCGCTGACGTGAACAAAGCCGACAGGTACGGCTTTACGCCGTTACATTTCGCCGTAGAAAGGGGTAACTTTGAGGTGGTAGATACACTGATCAAGAGAGGGGCCGACGTCAATCGAGTCGGTAGAAGAGGACACACGGCTTTGCACTGGGCTATCCGCGAAAATAATATCCAGGTGGTTAATGCTTTAATTGAAAGAGGTGCCGATGTAAATCGTGTTGGTCGCAGAGGACATACCGCTCTCCACTGGGCCATCAGGGAGAACAACATACAAGTCGTTAACGCACTCATCGAGAGGGGAGCGGACGTCAACCGAGTTTCTAGGTCTGGACATACAGTATTGCACTGGGCCGTAAAGTCAAACAACATTCAGGCCGTGAACACTCTGATTGAGCAAGGTGCCGAAATAAAAGACGTTGACTACCCGTTAGATTGGGCCGTAGGCTACGAGTTCCAGGATATTGCCAAACTCTTGATGGAGCACGAAGTCAAGTTAAAGGGCTGCAACGTGCGTAAACCTAGGGACATTGTACAACATAACCCAGACCTGGAGATCGAAGCGAGCGAATATATTGATCGGTGCGTGAACGAAATAGAAGGCATAAAGAAAGAAATTATAAATGGTAATGTCACTTTCTACGATCTCCTCACATCCAAAGGCGATAAACTAGCGAGCCTGATGCTGAACAAGGACATTGTCACGGTTCTCGAAAAAGGTGAATACAAATCAAAATTTCCCACCTATAGTAAGATTATGGAAAGTCAGTATGAGATTGGCAAAGAAAACCTTCACCTCTTCAATCGCGCTCTAGAAGCCATCTTCAATCTTTTTGTGGTTAAGAACCTACCTCTGGAAATAGGTGAAATGATTGTCAAATACTTACCGAGCTCCTTCGATCGGAAAAGCCTCATCAAACTGGTTGAAGTTAAGGGATTGCCCAACGAGAAGGTTATCGAGAGGCGTTCATCTCTAGAACACAAAACAATCCCAACCACCCAGACAGTCCAAGATATCCTCACGCAGATCAGGGGTATTCTGGACAATCAGCAGATCGAAGTCAACGACTTACTGACCCTCGAGTTCGCTACATCAGCGGGATACTACGATTATTGGCTTCAACAGATAGATATTGCCCGCGCAGCGCGCTTGCTGTACCAATTTTGCACTAAGGGTAACCATACGTTCGAGGTGGCGAACTTAGAGGGAAACGACGGAAACGAACCAATCGTGGGTGTGCTCAATCAATTCCGGGAAAACGAGGAACAACAAAGGTTGACCTTGATAATCACCCTAAACAACGCACACTGGGTAACTCTTGTTATCGAGCGTCAGAACGGGAATTATGTGGGCTATTATGCCGACTCCACTGCCACAGCCGTGCCAGGAGACATTACGGACATTATTCAAAACAACCTGGGAAATAACATCCGCATTAACAACGTATCAGTTTCCCAACAAACGGATGGATGGAACTGTGGCTTGTGGGCTCTTGAGAATGCCAACAGCATTAACAGAGTGTTGAATGAAAATCCAGTCGGTAAGGTCCAAAATATAATTAATATCATCCGAGACTTCTTGGAGAGAGGACATCCGAAGCGTGATAGGAACTACTTCCAAAATATCAGAGTTGGCATCTCACAACTATTCAGAAATGACCCCGGATTTCAGGATGTCCAATTGCAGGCATATATCCAAAACAGAGAGCAAATAGACCCCCTGTTGCGCACATACTTAGAACTTGGATACCATAGAGTGGGTGGGGGTTATGGTCTGGTCGAGGCCCCGATCAGTTTGGGTGTGTCGGATCCGAGTAATTTCGGTGACTTGCTGCAGTCTTCGCTGGAAGGCCCCGAGGCCTCTCGCGTGTCTGATCTTTCCAAAGGAAAAGGCGGCAGA

>wmk1 (original)

ATGGCAAACATCTCAATAAGGTATCAGATAGCACAAAAAGTAAGGAACTGGAGGTTAAAGCGAGGATATACTCAAAAAGATTTAGCGAAGAAAGTCAGTGTAACGTATCAAGTAGTACTACAATATGAAAAAGGAACACGTAAAATTTCGATTGAAAAGTTGTATGCTATAGCAGAGATATTGTCGATTGGTATTGTAGACCTAATTCCTGTATCAAATGAAAAAATCTGTCTTGAAGATGAGGGAGAAGAAATACTAAATCTAGTAAGAGAATATAAAAAGATTAATGATCAGGAATTACGTAAGATGTTTTGTTTGCTAACCAAATTTGTCCAAGTCAGTGAAAGAAGTAGTAGGAAAGCAGAGAAAGTAAAAATTGCAAAGGGTCTGGTTAAAGCAGGAGTTTCTGTTGATACTGTTTCAAAAACAATTGGTCTATCTGCTGATGAATGTGTTGAAGAAAAAACGGGTTCTATCTACTACCAAATAGGCAAAAAGATAAAGGAATGGAGACTAGTGAGAGAGTATACTCAAAAGGATTTAGCGGAGAAAATGAGTACAACACGTGACGAAATAAGCAACTATGAACAAGGACGCGTAGCTACTCCACTGGGAAAATTATATGAGATAGCAGAAACATTATCGATTAGTATTACAGATCTACTAACAGAGGAAGATGAAGGTAGTAAAGTGGAAAATGAGCTACCGAATTTGATTAAAGAATACAAAGAAATTGAGAGCCAAGAACTACGTCATGCACTAATAAAATCTTTGTTTGAAGGCATACGGATTTGCGAAGAAAAAGTGAAAAGAGCAGAAAAGATCAAAATTGCAAAAGACTTAGTAAAAAAAGGAATTTCTATCAATATTATTTTAAAAACAATAGGCATCTCTTTAGACGAAATTCAACAAATTTAA

>wmk1 (codon-optimized)

ATGGCCAACATTTCCATTCGCTATCAGATAGCTCAAAAGGTCAGGAATTGGCGTTTAAAGAGGGGTTATACGCAGAAAGATCTTGCGAAGAAAGTTAGCGTCACATATCAGGTCGTATTACAGTACGAAAAAGGAACACGGAAAATCAGCATTGAGAAGCTGTATGCTATAGCCGAAATCCTTTCCATTGGAATTGTCGATCTGATCCCAGTAAGCAATGAGAAGATATGCCTAGAAGACGAAGGTGAAGAGATACTAAACCTTGTTCGCGAGTACAAGAAAATCAATGACCAAGAACTGCGTAAGATGTTCTGTTTGCTCACCAAATTCGTCCAAGTTAGCGAAAGATCATCACGCAAAGCTGAGAAGGTGAAAATTGCCAAGGGATTGGTGAAAGCAGGTGTTTCTGTTGACACTGTTTCTAAAACCATCGGGTTAAGTGCGGATGAGTGTGTAGAGGAAAAAACAGGGTCTATCTACTACCAGATAGGCAAGAAGATAAAAGAGTGGAGGTTAGTGCGTGAATACACGCAAAAGGATCTGGCGGAGAAGATGAGTACTACTCGAGACGAAATCTCGAACTATGAACAAGGCAGAGTGGCAACTCCTTTGGGTAAACTCTACGAAATTGCTGAAACGCTCTCTATCTCCATAACCGACTTGTTAACCGAAGAAGATGAAGGAAGTAAAGTGGAAAACGAACTACCGAATCTGATCAAAGAGTACAAGGAAATCGAGTCACAAGAGTTGAGACACGCACTCATCAAATCACTTTTTGAGGGTATACGAATATGCGAAGAGAAAGTGAAAAGAGCCGAAAAAATCAAGATCGCTAAAGACCTGGTAAAGAAAGGCATATCGATTAACATTATCCTCAAGACAATTGGCATTTCGTTGGATGAGATTCAACAGATATAA

>wmk2 (original)

ATGGCAAATATCTCTACAAGGTATCAGATAGCAGAAAAAGTAAGGAGCTGGAGGTTAAAGCGAGGTTATACTCAAAAAGATTTAGCGGGAAAAATCGGCGTAACGTATCAAATAGTACTACAATATGAAAAAGGAACACGTAAAATTTCGATTGAAAAGTTGTATGCTATAGCAGAGATATTGTCGATTGGTATTATAGACCTAATTCCTGTATCAAATGAAAAAATCTGTTTTGAAGATGAGGGAGAGGAAATATTAAATCTAGTAAGAAAATATAAAAAGATTAATGATCAGGAATTACGTAGAATGTTTTGTTTACTAACCAAATTTGTTCAAGTTAGTGAGAAAAATAGTAAAAAAACGGAGAGAATAAAAATTGCAAAAGGTCTGGTTAAAGGAGGAGTTTCTGTTGATATTGTTGCAAAAACAATTGGTCTCTCTGCTGATGAATGTGTTGAAGAAAAAACGGGTTCTATATACTGCAAAATAGGGAAAAAGATAAAGGAATGGAGGGCAGTACAGGAATATACTCAGAAGGATTTAGCAGAGAAAATGAGTACAACACGTGACGAAATAAGCAATTATGAGCAAGGAAGAGTTGCTGTTCCACTTGATAAATTATATGCAATAGCAGAAACATTATCGATTAGTATTACAGATCTTCTAATAGAGGAAGGTAGTAAAGTAGAAAATGAATTACCGAATTTAATTGAAGAATACAAAAAGATTGAAAGCCAAGAACTACGTAATGTACTAATAAAATCTATGTTTAAAAGCATACAAATTTGTGAGGAGAAAATTAGAGAAGAAGAAAGAATCAAAATTGCAAAGAACTTAGTTAAAAAAGGAATTTCTACCGATATTATTTTGCAAATAACAGGCCTCTCTTTAGGCGAAATTCAACAAATTTAA

>wmk2 (codon-optimized)

ATGGCGAACATAAGTACTCGTTACCAAATAGCCGAGAAGGTCAGAAGTTGGCGACTTAAACGCGGATATACCCAGAAAGATTTGGCTGGGAAGATAGGTGTAACGTATCAGATCGTTCTCCAATACGAGAAAGGTACTCGCAAAATTTCGATTGAGAAATTGTACGCTATCGCGGAAATCTTGTCGATTGGCATCATAGACCTGATACCAGTGTCAAACGAGAAGATTTGCTTTGAAGACGAAGGTGAAGAAATCCTGAACTTAGTGCGGAAATACAAAAAGATTAACGACCAAGAATTACGTAGAATGTTTTGCCTACTTACCAAATTCGTGCAAGTAAGCGAAAAGAACTCCAAAAAAACCGAGCGTATCAAAATAGCTAAGGGACTGGTCAAAGGAGGCGTTTCTGTGGATATTGTAGCCAAGACGATTGGACTTTCTGCTGATGAGTGTGTGGAAGAGAAAACAGGTTCTATCTACTGCAAAATTGGCAAGAAGATAAAGGAATGGAGAGCTGTTCAGGAATACACACAGAAAGACCTAGCAGAGAAGATGTCTACAACTAGGGATGAAATCAGCAATTATGAACAAGGTAGAGTTGCAGTTCCTCTCGATAAGCTCTATGCCATAGCAGAGACTTTGTCGATTAGCATCACCGACTTACTGATCGAAGAAGGGTCAAAAGTCGAAAATGAACTTCCGAATCTGATAGAGGAATACAAGAAGATTGAGTCCCAAGAACTCCGCAATGTCCTCATCAAATCCATGTTCAAGAGTATTCAGATATGTGAGGAGAAGATACGAGAGGAAGAGAGGATAAAGATCGCCAAAAATCTGGTCAAAAAAGGCATTTCAACGGACATCATCTTGCAGATTACAGGACTATCATTAGGCGAAATTCAGCAAATCTAA

>wmk5 (original)

GTGGAAAAAAGTCTAGATTATAAAGTGGGAGAAAAATTAAAAAGCTGGAGGTTAGAGCGAGGGTACACTCAGAAAGATTTAGCAGAGAAAATTGGTGTAAAATACTGGGTGATATTGCAATATGAGAAAGGGAATCGTAGAGTTCCAATTGAAAGGTTGTATGCTATAACTGAGGCACTATCAATCAGTATTACGGATCTTATTCCTGTATCAAAAAGCTGTCTTGAAGATGAAGAAGAAATATTAAATCTAGTAAGAGAATATAAAAAGATTAACGATCAGGAGTTACGTAAGATGTTTTGTTTGCTAACCAAATTTGTCCAAGTTAGTGAGAAAAGTAGTAGAAAATCGGAAAAAATAAAAATTGCAAAGAGTTTGGTTAAAGCAGGAGTTTCTGTTGATATTGTTGCAAAAACAATTGGTCTGTCTGCTGATGAATGTGTTGAAGAAAAAACGGGTTCTATCTACTACCAAATAGGCAAAAAGATAAAGGAATGGAGACTAGTGAGAGAGTATACTCAAAAGGATTTAGCGGAGAAAATGAGTACAACACGTGACGAAATAAGCAACTATGAACAAGGACGCGTAGCTACTCCACTGGGAAAATTATATGAGATAGCAGAAACATTATCGATTAGTATTACAGATCTACTAACAGAGGAAGATGAAGGTAGTAAAGTGGAAAATGAGCTACCGAATTTGATTAAAGAATACAAAGAAATTGAGAGCCAAGAACTACGTCATGCACTAATAAAATCTTTGTTTGAAGGGATACGTATTTGTGAGGAGAAAGTGAGAGAGATAGAAAGGATTAAAGTTGCAAAGGATCTAGTAAAAGGGGGAATTTCTATTGATACTATTTTGCAAGCGGTAGGTTTATCTATTGATATGGTTTTAGATGGATAG

>wmk5 (codon-optimized)

ATGGAAAAATCGCTCGACTATAAGGTGGGTGAAAAACTGAAGTCCTGGAGATTAGAAAGAGGCTATACGCAAAAGGATCTAGCTGAGAAGATTGGGGTGAAATATTGGGTAATCCTTCAGTACGAGAAAGGAAATCGTAGAGTTCCCATTGAAAGGTTATACGCCATAACTGAAGCTCTCAGCATCTCGATTACAGACCTTATTCCAGTGTCGAAAAGTTGCCTTGAAGATGAGGAAGAAATCCTCAATCTTGTCAGGGAGTACAAGAAGATTAACGATCAGGAGTTACGCAAAATGTTCTGTCTGCTGACGAAATTCGTCCAAGTCTCAGAGAAAAGTAGCCGGAAATCCGAGAAGATCAAAATAGCCAAATCTTTGGTTAAAGCCGGAGTTTCAGTCGACATTGTTGCCAAGACAATTGGACTCTCTGCAGATGAATGCGTTGAGGAAAAGACTGGTTCCATATACTACCAGATAGGCAAAAAGATCAAAGAGTGGCGATTAGTGCGTGAATACACACAGAAAGACTTGGCGGAAAAAATGTCTACCACTAGAGATGAGATCAGCAACTATGAACAAGGACGTGTTGCGACTCCTTTGGGCAAGTTGTACGAGATTGCAGAAACGCTTTCTATATCCATTACCGACCTCTTGACAGAAGAAGATGAAGGGAGTAAGGTCGAAAATGAGCTACCGAACCTAATCAAGGAGTATAAGGAGATAGAAAGCCAAGAACTGCGACACGCTCTCATCAAGTCACTGTTTGAAGGTATCCGCATTTGTGAGGAAAAAGTGAGGGAAATAGAGCGCATCAAAGTGGCAAAAGACCTGGTAAAGGGTGGAATCTCGATAGATACCATCTTGCAAGCTGTAGGTCTGTCAATAGACATGGTATTAGACGGCTAA
