# Supplementary material for: A Wolbachia factor for male killing in lepidopteran insects
Source: Nat Commun. 2022 Nov 14;13:6764. doi: 10.1038/s41467-022-34488-y (PMC9663696; doi:10.1038/s41467-022-34488-y)
Supplement: Supplementary file 6 — Reporting Summary [file 41467_2022_34488_MOESM6_ESM.pdf]

nature portfolio

Corresponding author(s): Susumu Katsuma

Last updated by author(s): Oct 25, 2022

Reporting Summary

Nature Portfolio wishes to improve the reproducibility of the work that we publish. This form provides structure for consistency and transparency in reporting. For further information on Nature Portfolio policies, see our Editorial Policies and the Editorial Policy Checklist.

Statistics

For all statistical analyses, confirm that the following items are present in the figure legend, table legend, main text, or Methods section.

n/a	Confirmed	
☐	☒	The exact sample size (*n*) for each experimental group/condition, given as a discrete number and unit of measurement
☐	☒	A statement on whether measurements were taken from distinct samples or whether the same sample was measured repeatedly
☐	☒	The statistical test(s) used AND whether they are one- or two-sided<br>*Only common tests should be described solely by name; describe more complex techniques in the Methods section.*
☒	☐	A description of all covariates tested
☐	☒	A description of any assumptions or corrections, such as tests of normality and adjustment for multiple comparisons
☐	☒	A full description of the statistical parameters including central tendency (e.g. means) or other basic estimates (e.g. regression coefficient) AND variation (e.g. standard deviation) or associated estimates of uncertainty (e.g. confidence intervals)
☐	☒	For null hypothesis testing, the test statistic (e.g. *F*, *t*, *r*) with confidence intervals, effect sizes, degrees of freedom and *P* value noted<br>*Give P values as exact values whenever suitable.*
☒	☐	For Bayesian analysis, information on the choice of priors and Markov chain Monte Carlo settings
☒	☐	For hierarchical and complex designs, identification of the appropriate level for tests and full reporting of outcomes
☒	☐	Estimates of effect sizes (e.g. Cohen's *d*, Pearson's *r*), indicating how they were calculated

Our web collection on statistics for biologists contains articles on many of the points above.

Software and code

Policy information about availability of computer code

Data collection	Image Lab 6.0 (Bio-Rad), FLoid Cell Imaging station(Life Technologies), Leica Application Suite X Version 4.4.0.24861 (Leica), StepOne Software v2.3 (Thermo Fisher Scientific)
Data analysis	pae2png.py script (https://github.com/CYP152N1/plddt2csv), AlphaFold v.2.0 (full_dbs, max_template_date 2021-07-14), Canu v.2.1, BWA v.0.7.17, Pilon v.1.23, PGAP v.2021-05-19.build5429, ClustalW v.2.1, IQ-TREE v.2.2.0.3, BLASTn v.2.7.1, v2.9.0, v.2.12.0, tBLASTn v.2.12.0, BLASTp v.2.9.0, Skyline v.20.2, Proteome Discoverer v.2.4 (Thermo Fisher Scientific), Prism 9 (GraphPad), Image Lab 6.0 (Bio-Rad), Leica Application Suite X Version 4.4.0.24861 (Leica), SMART (a Simple Modular Architecture Research Tool, http://smart.embl-heidelberg.de), StepOne Software v2.3 (Thermo Fisher Scientific)

For manuscripts utilizing custom algorithms or software that are central to the research but not yet described in published literature, software must be made available to editors and reviewers. We strongly encourage code deposition in a community repository (e.g. GitHub). See the Nature Portfolio guidelines for submitting code & software for further information.

Data

Policy information about availability of data

All manuscripts must include a data availability statement. This statement should provide the following information, where applicable:

- Accession codes, unique identifiers, or web links for publicly available datasets
- A description of any restrictions on data availability
- For clinical datasets or third party data, please ensure that the statement adheres to our policy

Source data are provided as a source data file. The Oscar sequence, 40.5 kbp-long wFur genome sequence around the Oscar locus, and wFur genome have been deposited in GenBank under accession numbers LC657087, LC659497, and CP096925, respectively. SfMasc, SfdsxM, SfdsxF1, and SfdsxF2 have been deposited in

GenBank under accession numbers LC716474, LC716647, LC716648, and LC716649, respectively. The MS proteomics data have been deposited in the ProteomeXchange Consortium via the jPOST partner repository with the data set identifier PXD034837. All other data are included as Supplementary Information and Supplementary Data.
The public data used are as follows: O. furnacalis protein data (GCF_004193835.1_ASM419383v1_protein.faa [https://ftp.ncbi.nlm.nih.gov/genomes/all/GCF/004/193/835/GCF_004193835.1_ASM419383v1/]), genome assembly of O. furnacalis (GCA_004193835.1 [https://www.ncbi.nlm.nih.gov/data-hub/genome/GCF_004193835.1/]), Refseq representative prokaryotic genomes database (ref_prok_rep_genomes, [https://ftp.ncbi.nlm.nih.gov/blast/db/]), translated protein sequences of wPip (AM999887 [https://www.ncbi.nlm.nih.gov/nuccore/AM999887.1]), the wMel genome sequence (AE017196.1 [https://www.ncbi.nlm.nih.gov/nuccore/AE017196.1]).

Field-specific reporting

Please select the one below that is the best fit for your research. If you are not sure, read the appropriate sections before making your selection.

[x] Life sciences   [ ] Behavioural & social sciences   [ ] Ecological, evolutionary & environmental sciences

For a reference copy of the document with all sections, see nature.com/documents/nr-reporting-summary-flat.pdf

Life sciences study design

All studies must disclose on these points even when the disclosure is negative.

Sample size	For WB, IP, ICC, RT-qPCR, and injection experiments, sample sizes were selected based on the data obtained in previous studies.
Data exclusions	No data were excluded.
Replication	Experiments were repeated or replicated as described in the figure legends. All experiments shown could be reproduced as described.
Randomization	No randomization was performed. Experiments were performed by treating samples and controls side by side under indicated conditions.
Blinding	Egg sexes are not visually distinguished. For other experiments, blinding was not performed. Wherever possible, unbiased experimental procedures and data analysis were performed.

Reporting for specific materials, systems and methods

We require information from authors about some types of materials, experimental systems and methods used in many studies. Here, indicate whether each material, system or method listed is relevant to your study. If you are not sure if a list item applies to your research, read the appropriate section before selecting a response.

Materials & experimental systems

n/a	Involved in the study	
[ ]	[x]	Antibodies
[ ]	[x]	Eukaryotic cell lines
[x]	[ ]	Palaeontology and archaeology
[ ]	[x]	Animals and other organisms
[x]	[ ]	Human research participants
[x]	[ ]	Clinical data
[x]	[ ]	Dual use research of concern

Methods

n/a	Involved in the study	
[x]	[ ]	ChIP-seq
[x]	[ ]	Flow cytometry
[x]	[ ]	MRI-based neuroimaging

Antibodies

Antibodies used	Primary antibodies:<br>anti-Oscar (custom produced by Eurofins Genomics: 1:1000–1:2000 dilution)<br>anti-actin antibody (Santa Cruz, sc-1616-R: 1:2000 dilution)<br>anti-GFP antibody (MBL, 598: 1:5000–1:10000 dilution)<br>Monoclonal ANTI-FLAG® M2 antibody produced in mouse (Sigma-Aldrich, F1804: 1:3000 dilution)<br><br>Secondary antibodies:<br>Goat F(ab')2 Anti-Rabbit IgG - H&L (AP), pre-adsorbed (Abcam, ab 98505: 1:5000 dilution)<br>Goat Anti-Mouse IgG H&L (Alkaline Phosphatase) preadsorbed (Abcam, ab 97032: 1:5000 dilution)<br>Goat anti-Mouse IgG (H+L) Secondary Antibody, HRP (Thermo Fisher Scientific, 626520: 1:5000 dilution)<br>Peroxidase AffiniPure Goat Anti-Rabbit IgG (H+L)(Jackson Immuno Research Laboratories, Inc., 111-035-144: 1:10000 dilution)<br>Alexa Fluor 488 F(ab')2 fragment of goat anti-rabbit IgG (H+L) (Invitrogen, 11070: 1:400 dilution)
Validation	anti-Oscar antibody was produced in rabbit by Eurofins Genomics, and verified using in vitro synthesized Oscar protein in this paper.<br>anti-actin antibody is produced in rabbit and validated by Santa Cruz (sc-1616-R)

anti-GFP antibody is produced in rabbit and validated by MBL (598)
Monoclonal ANTI-FLAG® M2 antibody is produced in mouse and validated by Sigma-Aldrich (F1804)

Eukaryotic cell lines

Policy information about cell lines

Cell line source(s)	BmN-4 (Bombyx mori, provided by C. Yasunaga-Aoki), Sf-9 (Spodoptera frugiperda, provided by R. Sato), OfT1A, OfT1B, OfT1C (in house, Ostrinia furnacalis). BmN-4 is available from RIKEN BRC. Sf-9 is available from Thermo Fisher, etc.
Authentication	Every cell line was authenticated by sequencing several genes (i.e. BmMasc, SfMasc, OfMasc etc.)
Mycoplasma contamination	Not checked.
Commonly misidentified lines (See ICLAC register)	No commonly misidentified cell lines were used in the study.

Animals and other organisms

Policy information about studies involving animals; ARRIVE guidelines recommended for reporting animal research

Laboratory animals	Bombyx mori (silkworm), eggs, female and male, p50T strain
Wild animals	Ostrinia furnacalis (corn borer moths), O. scapulalis, female and male, all developmental stages, collected at Nishi-Tokyo, Japan (35.7º N, 139.5º E) and Matsudo, Japan (35.8º N, 139.9º E), and cultured in our lab until their family lines fail.
Field-collected samples	O. furnacalis and O. scapulalis moths used in this study were collected at Nishi-Tokyo, Japan (35.7º N, 139.5º E) in early summer of 2020 and 2021. Wolbachia-infected O. furnacalis moths were collected at Matsudo, Japan (35.8º N, 139.9º E) in early summer of 2014 and at Nishi-Tokyo, Japan (35.7º N, 139.5º E) in early summer of 2021, and Wolbachia-infected O. scapulalis moths were collected at Matsudo, Japan (35.8º N, 139.9º E) in early summer of 2020. Larval O. furnacalis and O. scapulalis were reared on an artificial diet (Insecta LFS, Nosan Corp., Japan) at 25ºC under a photoperiod of 16L and 8D in the lab.
Ethics oversight	This study does not require the ethical approval.

Note that full information on the approval of the study protocol must also be provided in the manuscript.
